# Convoluted nasal passages function as efficient heat exchangers in ankylosaurs (Dinosauria: Ornithischia: Thyreophora)

**DOI:** 10.1371/journal.pone.0207381

**Published:** 2018-12-19

**Authors:** Jason M. Bourke, Wm. Ruger Porter, Lawrence M. Witmer

**Affiliations:** 1 Department of Biological Sciences, Ohio University, Athens, Ohio, United States of America; 2 Department of Biomedical Sciences, Heritage College of Osteopathic Medicine, Ohio University, Athens, Ohio, United States of America; Perot Museum of Nature and Science, UNITED STATES

## Abstract

Convoluted nasal passages are an enigmatic hallmark of Ankylosauria. Previous research suggested that these convoluted nasal passages functioned as heat exchangers analogous to the respiratory turbinates of mammals and birds. We tested this hypothesis by performing a computational fluid dynamic analysis on the nasal passages of two ankylosaurs: *Panoplosaurus mirus* and *Euoplocephalus tutus*. Our models predicted that *Panoplosaurus* and *Euoplocephalus* would have required 833 and 1568 thermal calories, respectively, to warm a single breath of air by 20°C. Heat recovery during exhalation resulted in energy savings of 65% for *Panoplosaurus* and 84% for *Euoplocephalus*. Our results fell well within the range of values for heat and water savings observed in extant terrestrial amniotes. We further tested alternate airway reconstructions that removed nasal passage convolutions or reduced nasal vestibule length. Our results revealed that the extensive elaboration observed in the nasal vestibules of ankylosaurs was a viable alternative to respiratory turbinates with regards to air conditioning. Of the two dinosaurs tested, *Euoplocephalus* repeatedly exhibited a more efficient nasal passage than *Panoplosaurus*. We suggest that the higher heat loads associated with the larger body mass of *Euoplocephalus* necessitated these more efficient nasal passages. Our findings further indicate that the evolution of complicated airways in dinosaurs may have been driven by the thermal requirements of maintaining cerebral thermal homeostasis.

## Introduction

**Fig 1:**
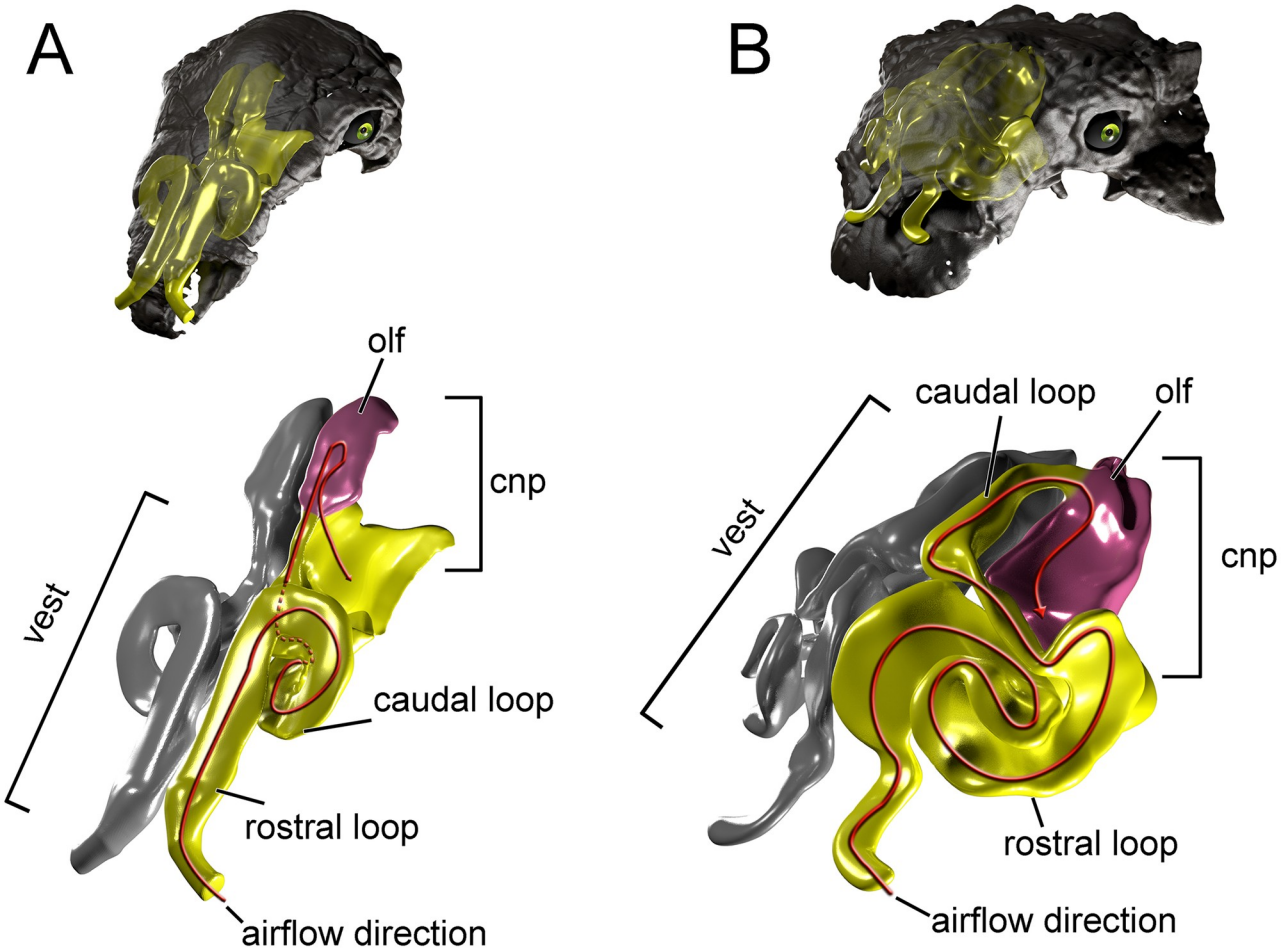
Cranium and nasal passage of the two ankylosaur species used for this study. The nodosaurid, *Panoplosaurus mirus*, ROM 1215 (A) and the ankylosaurid, *Euoplocephalus tutus*, AMNH 5405 (B). Non-modeled nasal passages are shown in greyscale.

Ankylosaurs were a successful group of ornithischian dinosaurs that had a near global distribution throughout the Cretaceous [1]. Ankylosaurs are best known for their well-armored hides, afforded by extensive osteoderm coverage across the back, sides, and tail, as well as the head [1–3]. In members of Ankylosauridae, these osteoderms continued into the tail where they ended in an expanded and ankylosed tail club. The process behind dermal ossification in ankylosaurs has attracted much interest over the years [4–7]. The mechanism behind the mineralization of soft tissues into a toughened, armored hide appears to have been somatically global, resulting in the mineralization of other epidermal and cartilaginous structures within the body, potentially including the eyelids in *Euoplocephalus tutus* [8]. This tendency to mineralize these soft tissues extended into the nasal passage where Maryańska [9] first observed bony or mineralized structures in the nose of *Pinacosaurus grangeri* that were typically cartilaginous or mucosal in other clades. She interpreted these structures within the nasal cavity of *P*. *grangeri* and other ankylosaurs as turbinates or conchae [9,10]. Brown [11] was perhaps the first to report the complexity of the nasal cavities of ankylosaurs, pointing to a symmetrical series of air spaces in the snout of *Ankylosaurus magniventris*. Coombs [12] later uncovered a similarly elaborate series of chambers within the nasal cavity of *E*. *tutus* which he interpreted as an extensive set of paranasal sinuses surrounding the nasal capsule [12–14]. Osmólska [15] suggested a rostral placement of the nasal gland in ankylosaurs based on a sectioned-off recess within the nasal vestibule of *P*. *grangeri*. Later work challenged this interpretation in favor of an enlarged paranasal sinus system [16] somewhat akin to that described by Coombs for *Euoplocephalus*. Similar observations of paranasal pneumaticity were suggested for other ankylosaurs as well based on the extensive excavations repeatedly uncovered within the nasal cavities of these dinosaurs [14,17–19]. It was only after a detailed computed tomographic (CT) scan of various ankylosaur skulls that it became evident that many of the structures initially interpreted as running parallel to a rather simplistic respiratory passage were in fact parts of bony laminae that braced and separated loops of a remarkably complicated nasal vestibule ([20,21], Fig 1).

Such a complicated structure begs for a functional explanation. The nasal passage in extant animals offers a variety of functions that amniotes have emphasized in different ways. The nasal passage is a large component of the conducting portion of the respiratory system [22], delivering air from the environment to the lungs. The nasal passage functions in modulating air coming into the lungs by filtering out dust and pathogens. Only a specific portion of the nasal passage functions in odorant detection. The requirements of olfaction run counter to the need to oxygenate the body, requiring regional separation of the nasal passage into a respiratory region and a slower moving olfactory region. The latter region may be expanded into a blind space referred to as the olfactory recess or olfactory chamber in macrosmatic species such as dogs and crocodylians [23,24]. The nasal passage offers a secondary function in phonation, providing resonance to sound waves coming from the pharynx and even acting as a primary means of sound production in certain animals such as saiga antelope and male gharials [25–27]. Lastly, the nasal passage functions to condition respired air by warming and humidifying it upon inspiration and then cooling and drying it on expiration. This conditioning capacity of the nasal passage received extensive study in the latter part of the 20^th^ century [28–33], especially in relation to respiratory turbinates or conchae and their association with the evolution of tachymetabolic endothermy [34–39].

Witmer and Ridgely [20] briefly suggested that the elongated airways of ankylosaurs may have functioned in thermoregulation or in vocal resonance. Miyashita et al. [21] expanded on this argument, offering evidence against an olfactory explanation and for either a thermoregulatory or vocal resonance function. Regarding the former, Miyashita et al. [21]described the extensive surface area that a looping nasal passage offers, and coupled this enhanced surface area with evidence for extensive vascular irrigation surrounding the elongated nasal vestibule [20,21]. Such a combination would have resulted in heat transfer from the body to the air regardless of whether or not this was the primary function of the nasal passage [21]. However, until now, the thermoregulatory function of these elaborate nasal passages had been inferred solely based on anatomy.

**Fig 2:**
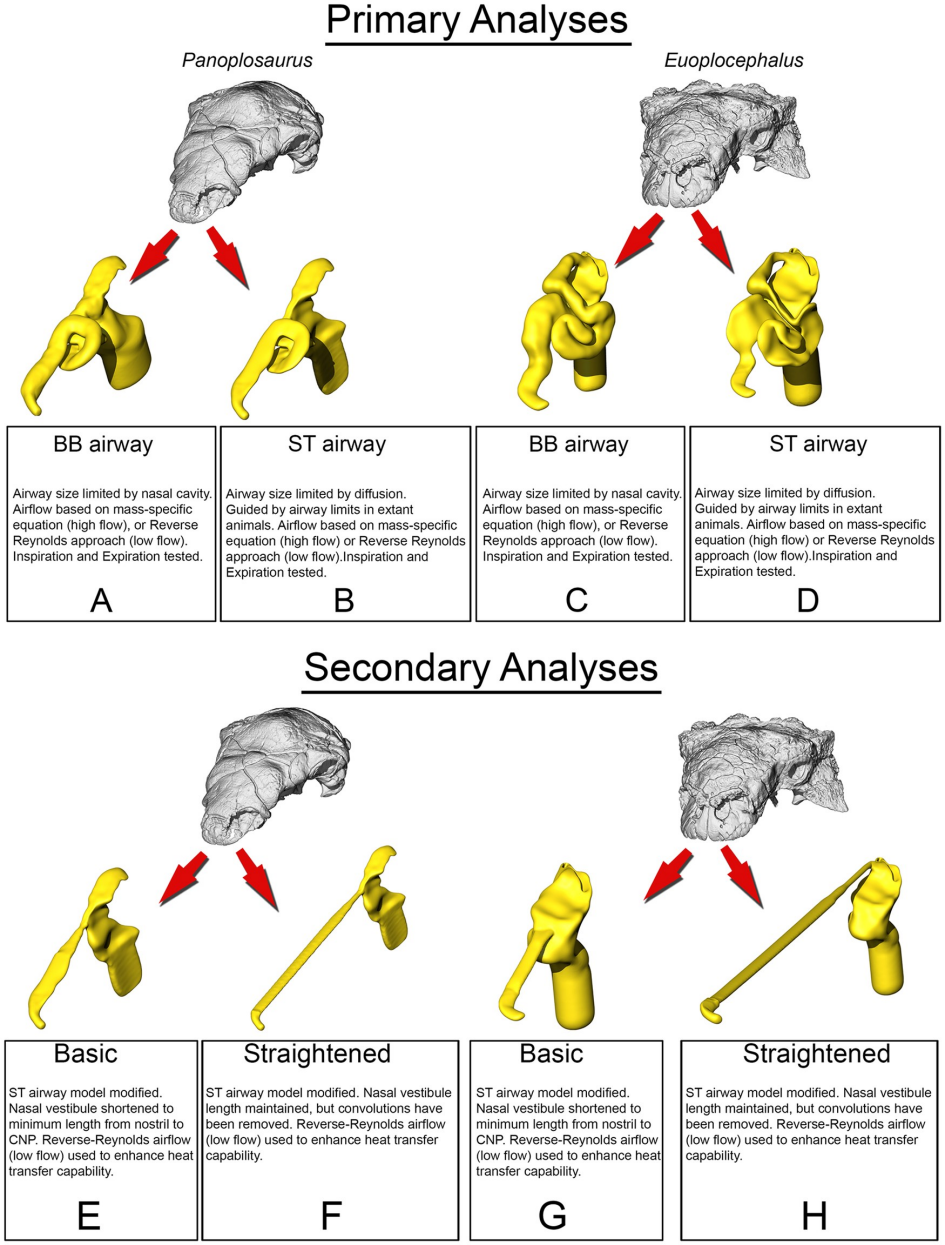
Summary of models analyzed in this study. *Panoplosaurus* (A,B,E,F) and *Euoplocephalus* (C,D,G,H) nasal passages were modeled as preserved, or bony-bounded (A,C), with soft-tissue correction (B,D), simplified (E,G) or with all convolutions removed (F,H). See Methods section for specifics for each model.

We tested the functional hypothesis that the nasal passage in ankylosaurs was an efficient heat exchanger by modeling the airways of two different ankylosaurs; a nodosaurid and an ankylosaurid. To simulate the flow of heat between the nasal passage and the air, we performed a computational fluid dynamic (CFD) analysis. Digital simulation of fluid movement via CFD is routinely performed in the fields of aeronautics [40], automobile engineering [41], and building ventilation [42]. CFD has been successfully applied to biological problems, especially in the realm of biomedicine [43,44], and has been successfully used to simulate airflow in the nasal passages of extant animals [45,46]. Using digital models alleviates the costs associated with physical models, such as choice of material, as well as the costs and complications associated with physical experiments such as flume systems and wind tunnels. Digital simulation of fluid movement via CFD provides a cost-effective means of testing multiple flow speeds, which is difficult to obtain *in vivo* for extant animals and impossible for long-extinct taxa. An added benefit of digital simulations is that they provide the opportunity to digitally manipulate the nasal passages which allowed us to test the preserved airways against soft-tissue-corrected versions. This approach allowed us to perform secondary analyses that tested the effects of length and convolutions using digitally manipulated airways that were either shortened or straightened (Fig 2). In addition to general airflow characteristics, we modeled the heat-transfer potential within the nasal passages of these two dinosaurs. General energetic costs associated with heating and humidifying the airway were calculated based on equations from modern birds. Results were compared to each other and to previously published results on extant animals to determine if the effectiveness of the nasal passage in ankylosaurs was within the range observed in extant animals. We were particularly interested in seeing how well a non-turbinate filled nasal passage would compare to the extensive, turbinate-filled noses of most extant mammals and birds.

To ensure that our methods were sound, we performed a validation experiment using a digitized pigeon airway and compared our results to previously published results in the literature.

## Institutional abbreviations

AMNH, American Museum of Natural History, New York, USA; CMN, Canadian Museum of Nature, Ottawa, CA; ROM, Royal Ontario Museum, Ontario, CA; ZPAL, Institute of Paleobiology (Zaklad Paleobiologii) of the Polish Academy of Sciences, Warsaw, PL.

## Anatomical abbreviations

a nas, apertura nasalis; air, airway; ant sin, antorbital sinus; bone, bone of nasal cavity; bna, bony narial aperture; cap, cartilaginous nasal capsule; ch, choana; ch f, choanal fossa; ch fd, choanal fold; ch fp, choanal flap; ch g, choanal groove; cnp, cavum nasi proprium; co, concha; ept, ectopterygoid; f ex, fenestra exochoanalis; j, jugal; lam, lamina transversa; max, maxilla; mnca, median nasal caputegulum; mu, mucosa; nar, naris; ns, nasal; nvas, neurovasculature; olf, olfactory recess; olf turb, olfactory turbinate; p2, secondary palate; pal, palatine; pmax, premaxilla; pt, pterygoid; q, quadrate; tr, tracheal extension; turb, turbinate; v, vomer; vest, nasal vestibule.

## Results

### Validation test

**Fig 3:**
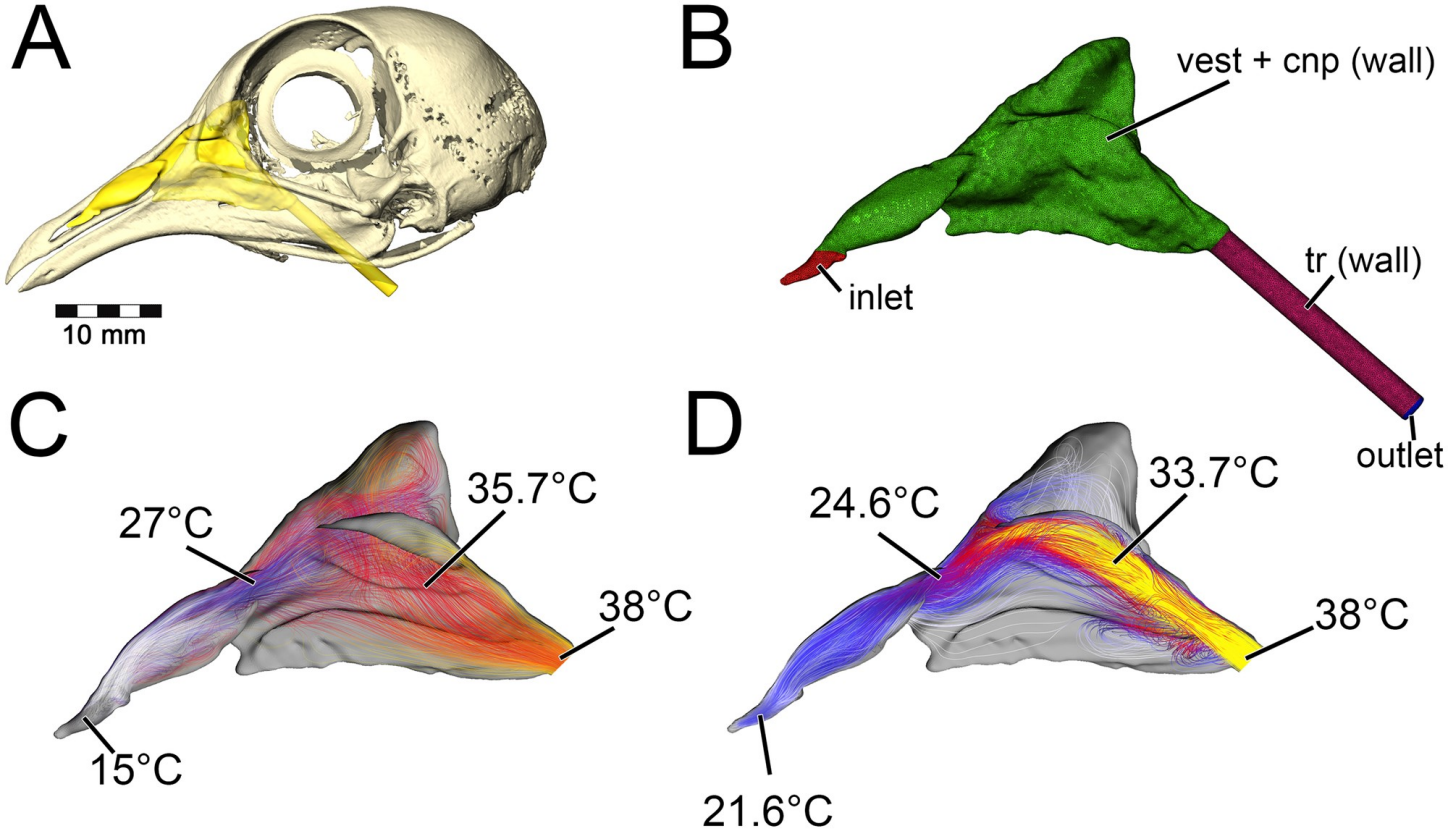
Airflow and heat transfer within the left nasal passage of a pigeon (*Columba livia*). (A) Airway was segmented out from the head and (B) converted into a volumetric mesh for CFD analysis following methods in the text. (C) Heat transfer simulation was performed under an inspiratory flow condition and data from that simulation was used to inform (D) the expiratory flow conditions. Artificial laryngotracheal extension was omitted in C & D as no data from that region was used.

During inspiration, the nasal passage of our pigeon model warmed incoming air by 22°C, bringing the air field close to body temperature by the time it reached the throat. Subsequently, a 22°C drop in temperature was applied to the nasal walls of the expiration model. To test heat transfer during expiration, air within the trachea was set to a temperature of 38°C, reflecting empirically obtained data on tracheal temperature in pigeons during expiration [38]. The converged expiration model revealed an air temperature drop from 38°C to 21.6°C at the nostril (Fig 3). This 16.4°C drop in temperature was within the range of values obtained by Geist ([38], Table 1), suggesting that our methodology was producing results similar to *in vivo* animal experiments.

**Table 1:** Comparison of values for heat transfer in domestic pigeons (*Columba livia*) between experimental data [38] and simulation (this study).

Study	Mass (g)	Body temp (°C)	Ambient air (°C)	Oral temp (°C)	Exhaled temp (°C)
38	319 (+/- 45.2)	40.7	15	38.2 +/- 0.5	21.4 +/- 0.5
This study	455	40.7	15	38	21.6

### Panoplosaurus mirus

#### Bony-bounded (BB) airway (Fig 2A)

**Fig 4:**
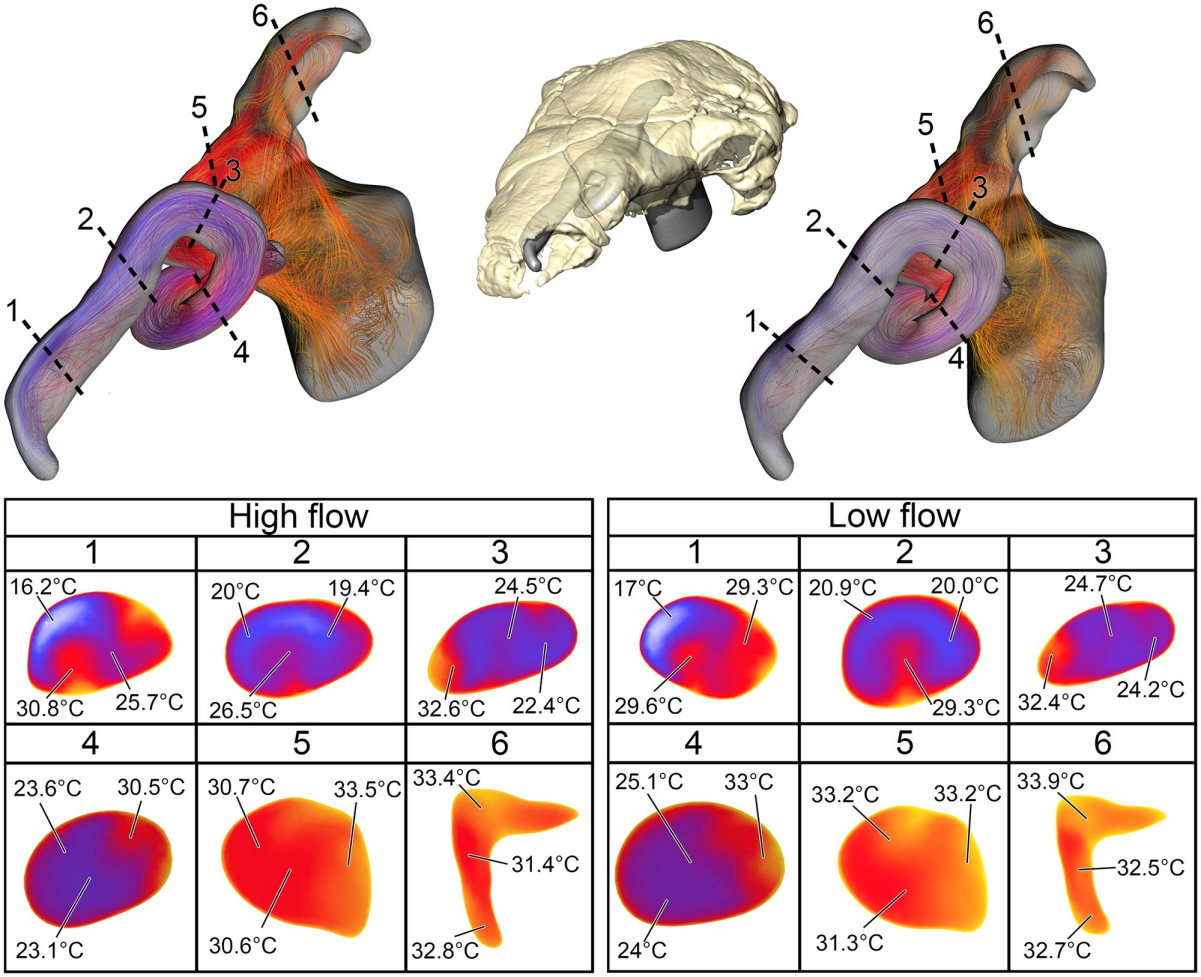
Heat flow within the BB nasal passage of *Panoplosaurus mirus* (ROM 1215) during inspiration under both high (left) and low (right) flow scenarios. Numbers of dotted lines indicate cross-section numbers. Cross sections were taken at equivalent locations on both models.

Under the high flow rate condition, the BB airway was able to successfully increase inspired air temperature by 18.2°C (Fig 4). Most (93%) of this heating took place inside the elongate nasal vestibule. The relative humidity of the inspired air reached saturation prior to entering the cavum nasi proprium (CNP). The larger volume of the nasal passage produced slower-moving air with a fair amount of vorticity present inside the CNP. Inspired air left the choana at 33.2°C. During expiration, air left the nostrils at 22.7°C. Under the low flow rate condition, the BB nasal passage was able to warm inspired air to 18.6°C, with 92% of airway heating taking place within the nasal vestibule. Moisture content of the air achieved saturation earlier than in the high flow condition. The low flow rate condition exhibited more laminar airflow compared to the more turbulent high flow condition. On expiration, the low flow condition BB airway reduced air temperature down to 20.5°C prior to exiting the nostrils.

#### Soft-tissue (ST) airway (Fig 2B)

**Fig 5:**
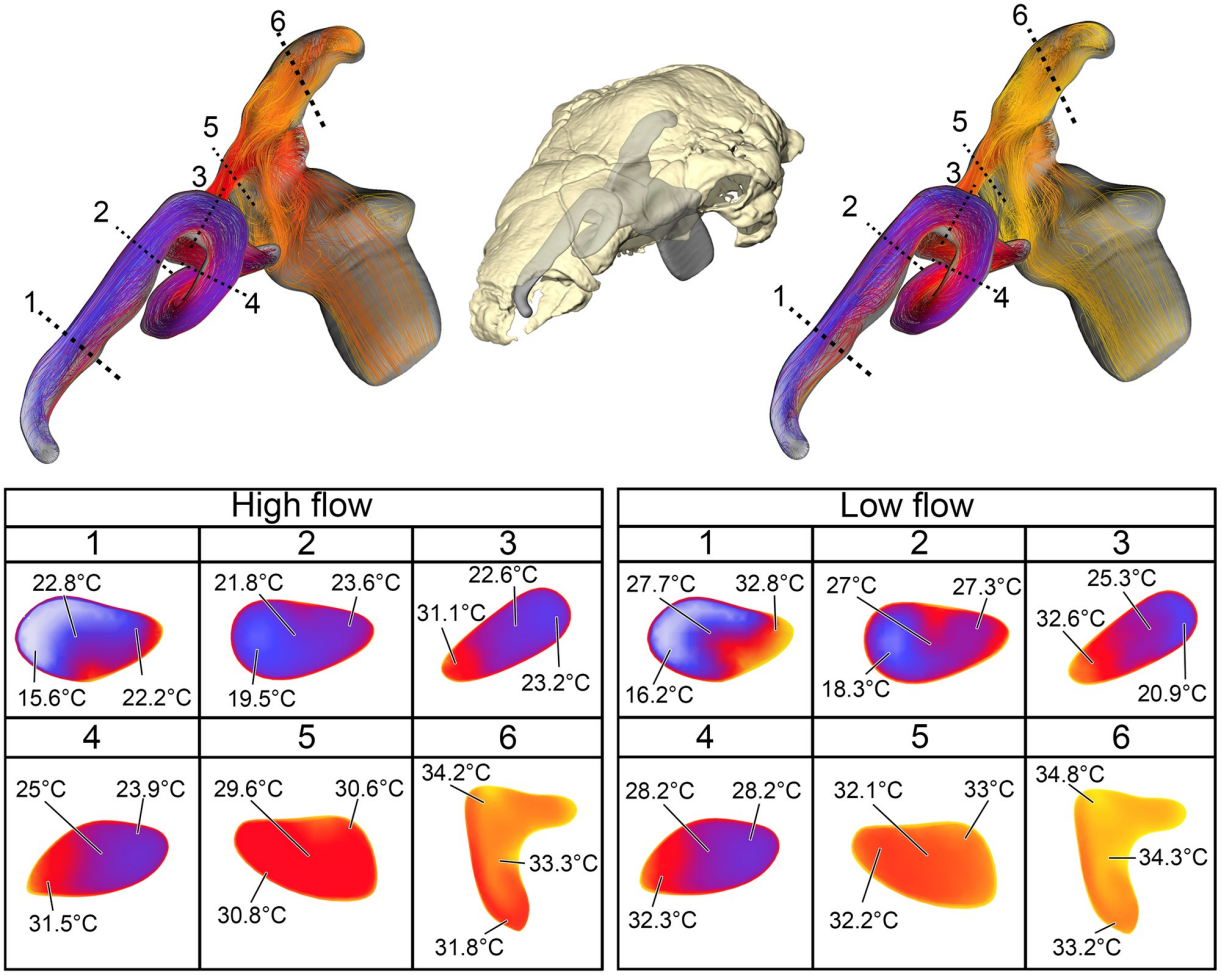
Heat flow within the soft-tissue corrected nasal passage of *Panoplosaurus mirus* (ROM 1215) during inspiration under both high (left) and low (right) flow scenarios. Numbers of dotted lines indicate cross-section numbers. Cross sections were taken at equivalent locations on both models.

Under the high flow rate condition, the nasal passage of *Panoplosaurus* was able to heat inspired air by 17.9°C. The convoluted nasal vestibule was responsible for the majority of the heat transfer (94%, Fig 5). Similarly, relative humidity of the inspired air had reached saturation prior to entering the CNP. Air left the choana at 32.9°C. Vortices were observed in the first portion of the nasal vestibule (rostral loop of Witmer & Ridgely [20]) as well as the CNP near the olfactory recess. Upon expiration, air entered the choana at 35°C and left the nostril at 21.6°C. Expiratory flow was more laminar than inspiratory flow, with very few vortices observed throughout the nasal passage. Under the low flow rate condition, the nasal passage of *Panoplosaurus* warmed inspired air by 19.3°C. Similar to the high flow rate condition, most of the heat transfer (89%), and all of the moisture transfer, occurred within the convoluted nasal vestibule (Fig 5). The CNP contributed more to airway heating under this scenario than under the high flow rate condition. Similar vortices were observed in the low flow rate condition as in the high flow rate condition (Fig 5). On expiration, air in the low flow rate condition left the nostril at 19.5°C.

#### Basic airway (Fig 2E)

**Fig 6:**
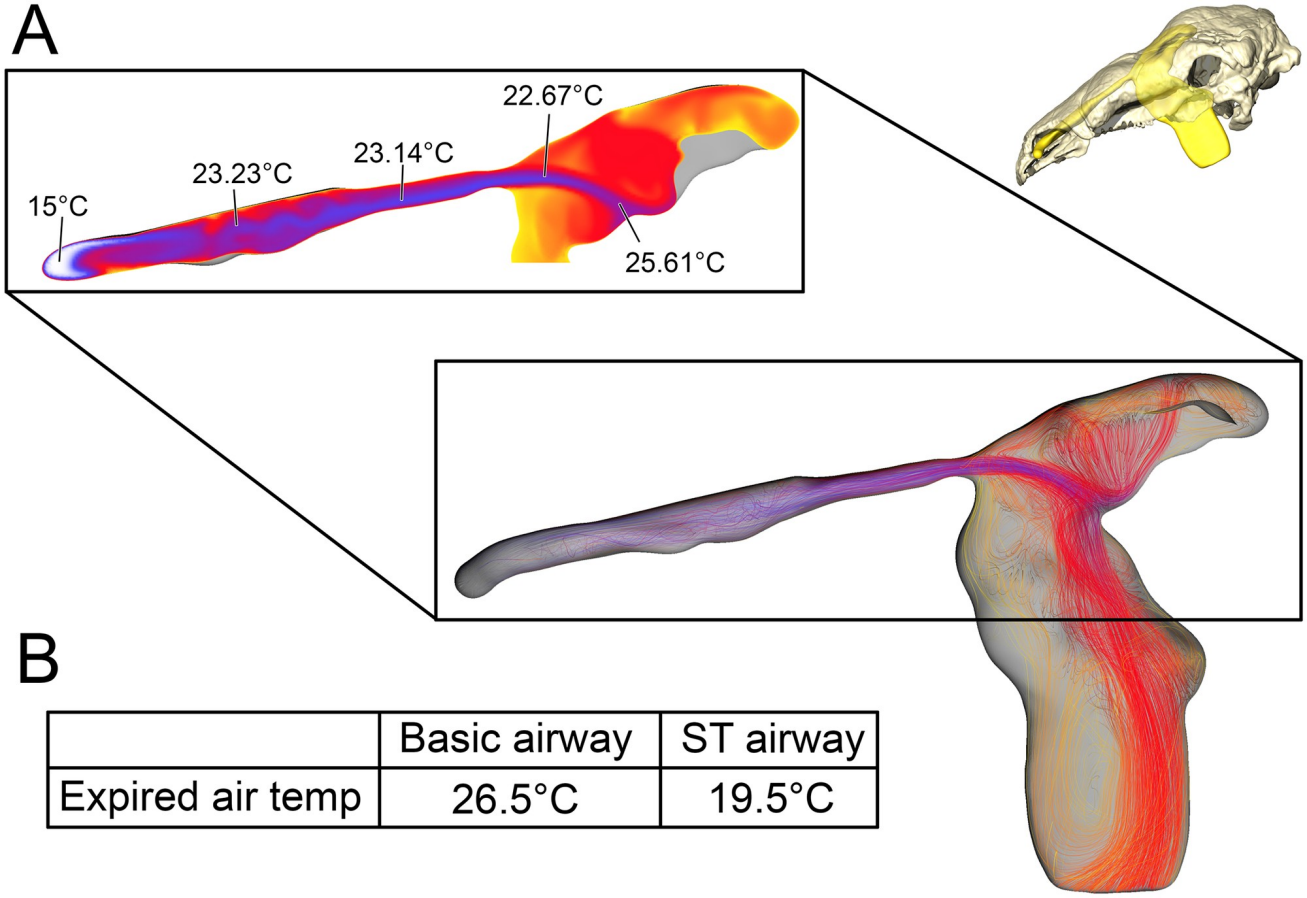
Airflow through the basic airway of *Panoplosaurus mirus* (ROM 1215). (A) Left lateral view of nasal passage with air streams showing general air field pattern. Airway is color-coded for temperature (hotter colors = hotter temperatures. *Inset*: Sagittal cross section of the nasal vestibule and CNP showing central stream of cool air passing through the nasal vestibule. (B) Temperature at the nostril during expiration for the basic airway and the ST airway.

The basic airway consisted of a plesiomorphic, truncated nasal vestibule that extended in a straight line from the nostril to the opening of the CNP (Fig 6). The total length of this simplified nasal vestibule was 200 mm. This reduced the length of the original 440 mm nasal vestibule by 55%. As this basic airway was strictly a hypothetical construct, we only ran the model under the more conservative, Reversed-Reynolds condition (see Methods). The lower flow rate associated with this condition provided the shorter airway with the best opportunity to transfer heat from the nasal passage. Despite this lower flow rate, the basic airway had difficulty transferring a substantial amount of heat from the nasal passage to the inspired air. On the outset, this difficulty was not entirely clear as the entire nasal passage was able to warm inspired air by 17.6°C (Fig 6) and achieve moisture saturation. However, only 63% of the heat exchange took place inside the truncated nasal vestibule. This was evident upon examining the temperature distribution through the nasal passage (Fig 6). Sagittal cross sections of the nasal passage revealed a consistent, low-temperature central stream of air that traveled through the nasal vestibule and remained largely unchanged by the surrounding nasal mucosa. This resulted in a cool stream of air entering the CNP (Fig 6A). Placing a greater emphasis on the CNP to heat the remainder of the air field proved detrimental to heat savings as the final expired air temperature at the nostril was a relatively high 26.5°C (Fig 6B).

#### Straightened airway (Fig 2F)

**Fig 7:**
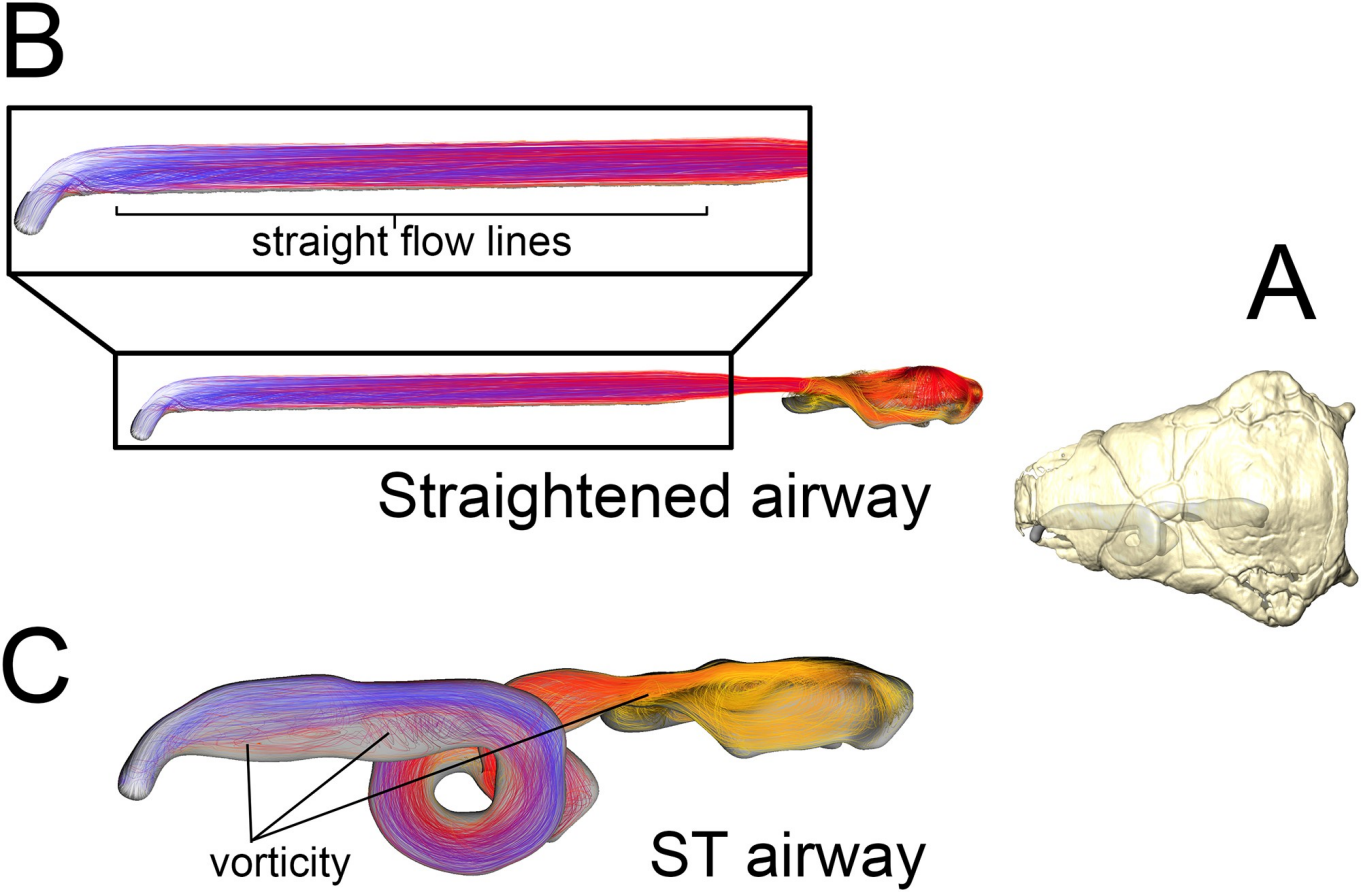
Airflow comparison between the straightened airway and the ST airway in *Panoplosaurus mirus* (ROM 1215). (A) Dorsal view of the skull of *P*. *mirus* with left ST airway in situ. (B) Dorsal view of the straightened airway with flow lines in place. Airflow lines are color-coded for temperature (hotter colors = hotter temperatures). *Inset*: Magnified region of nasal vestibule showing evenly spaced, straight flow lines. (C) Dorsal view of the ST airway under the low flow scenario. Vorticity is observable throughout the nasal vestibule. Note: ST airway in C is not to scale with straightened airway B.

Removing the curvature from the lengthened nasal vestibule ameliorated the vorticity observed in the BB and ST airway models. Under the low flow condition, the straightened airway warmed air by 18.3°C prior to leaving through the choana (Fig 7). 78% of that heating occurred in the much-elongated nasal vestibule. Similarly, the elongated vestibule was able to completely saturate the inspired air field prior to reaching the CNP, as in the ST and BB airway models. On expiration, the nasal passage reduced the heat of the expired air by 11°C, resulting in expired air leaving the nostril at 23.9°C.

### Euoplocephalus tutus

#### Bony-bounded (BB) airway (Fig 2C)

**Fig 8:**
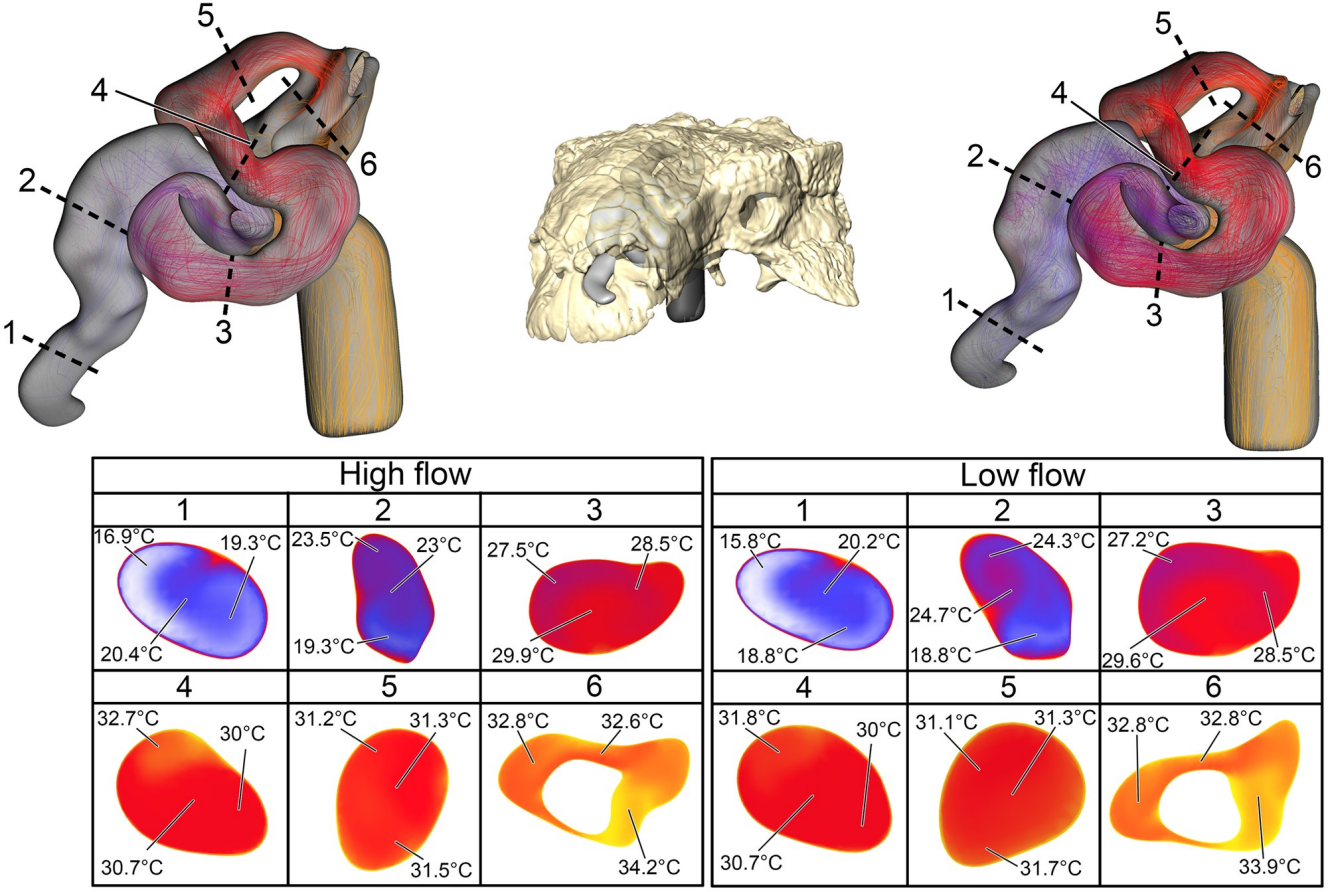
Heat flow within the BB nasal passage of *Euoplocephalus tutus* (AMNH 5405) during inspiration under both high (left) and low (right) flow scenarios. Numbers of dotted lines indicate cross-section numbers. Cross sections were taken at equivalent locations on both models.

Under the high flow rate condition, the BB airway was able to warm inspired air by 18.8°C, with 82% of that heating taking place within the confines of the nasal vestibule (Fig 8). Relative humidity of inspired air reached saturation by the time it reached the caudal loop of the nasal vestibule. Vorticity was evident in most of the bends of the nasal vestibule as well as inside the spacious CNP. On expiration, the nasal passage reduced the expired air temperature by 13.9°C, resulting in air leaving the nostrils at 21.1°C. Under the low flow rate condition, the BB airway warmed inspired air by 18.8°C, with 88% of the heat exchange and 100% of the moisture exchange occurring inside the convoluted nasal vestibule (Fig 8). Despite a lower flow rate, standing vortices along the curves of the nasal vestibule were still present. On expiration at this low flow rate, the nasal passage was able to reduce the temperature of expired air by 15.6°C, resulting in air leaving the nostrils at 19.4°C.

#### Soft-tissue (ST) airway (Fig 2D)

**Fig 9:**
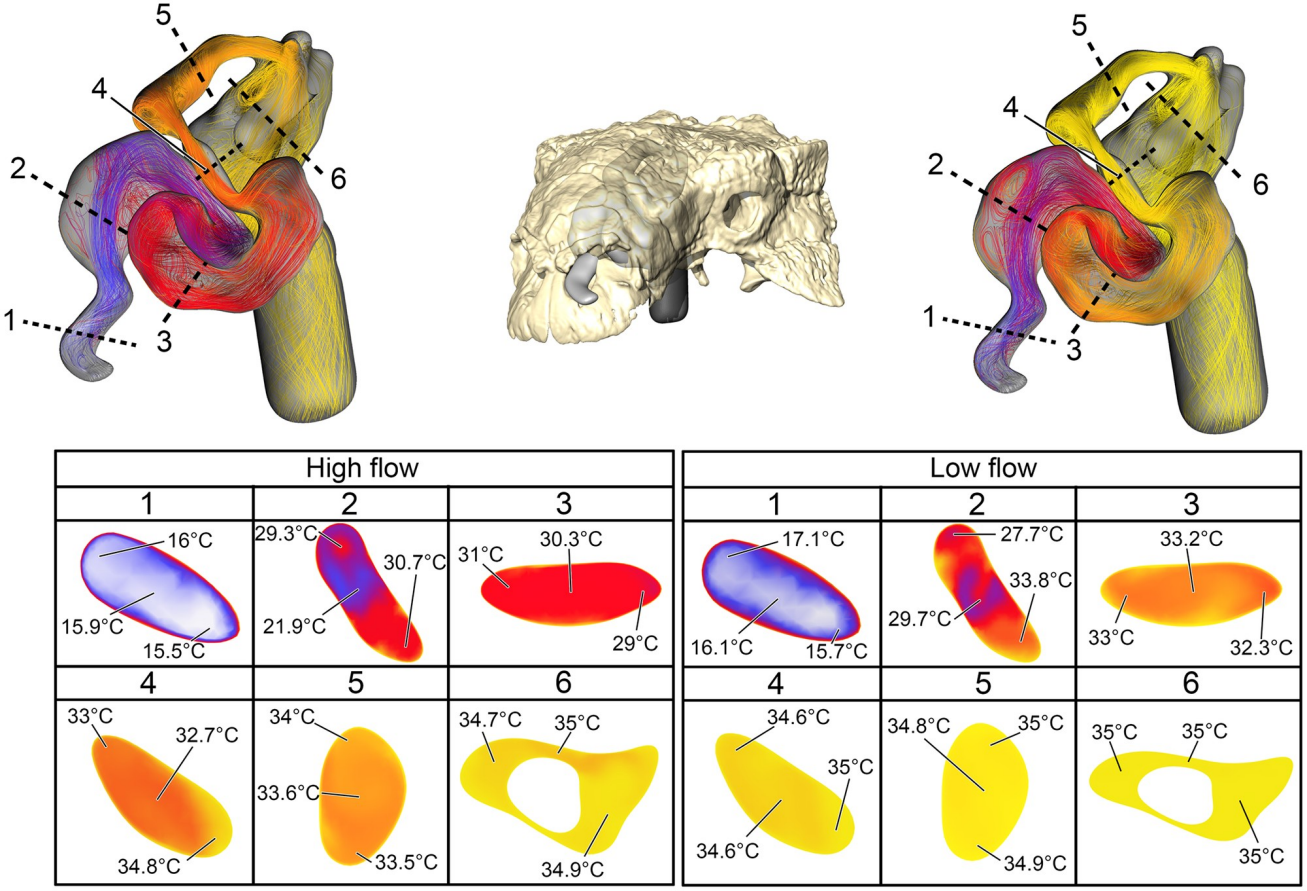
Heat flow within the soft-tissue corrected nasal passage of *Euoplocephalus tutus* (AMNH 5405) during inspiration under both high (left) and low (right) flow scenarios. Numbers of dotted lines indicate cross-section numbers. Cross sections were taken at equivalent locations on both models.

Under the high flow rate condition for *Euoplocephalus*, the nasal passage warmed air by 19.7°C with essentially all that heat transfer (97%) occurring in the convoluted nasal vestibule (Fig 9). Air field relative humidity reached saturation earlier in the nasal vestibule of the ST airway than the BB airway. Extensive vorticity was observed throughout the nasal passage. These vortices were often concentrated around the multiple convolutions within the nasal passage. Upon expiration, air entered the choana at 35°C and exited the nostril at 17.3°C. As with *Panoplosaurus*, there were fewer vortices upon expiration than inspiration. Under the low flow rate condition for *Euoplocephalus* the air field showed complete warming from ambient (15°C) to body temperature (35°C) with almost all of the heat transfer occurring within the convoluted nasal vestibule (99%). Relative humidity of the air field reached saturation slightly earlier within the nasal vestibule. Similar flow patterns to the high flow condition were observed under the low flow rate condition (Fig 9). During expiration, air left the nostril at 15.9°C, which was just above ambient temperature.

#### Basic airway (Fig 2G)

**Fig 10:**
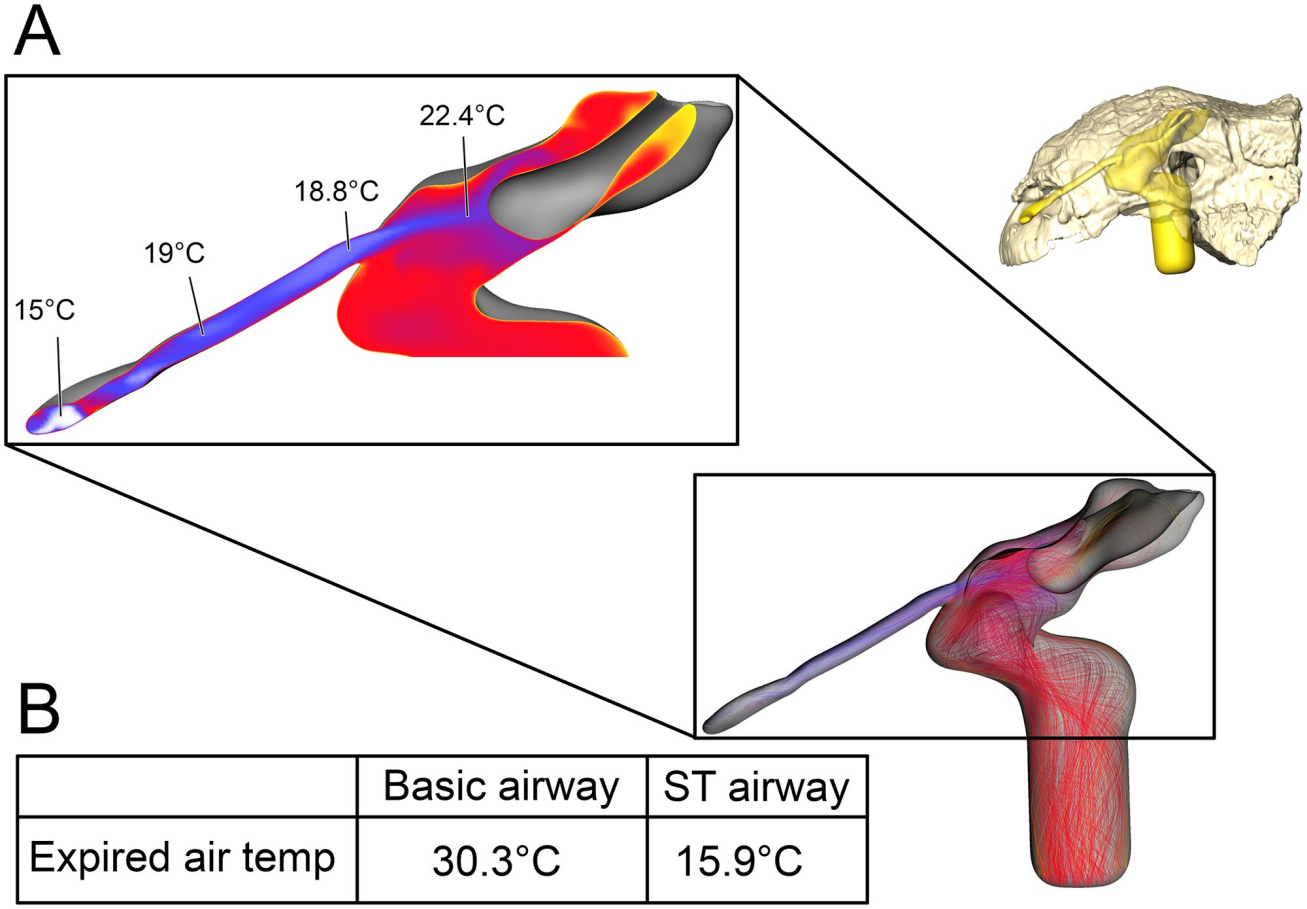
Airflow through the basic airway of *Euoplocephalus tutus* (AMNH 5405). (A) Left lateral view of basic airway showing airflow. Streamlines are color-coded for heat (hotter colors = hotter temperatures). *Inset*: Sagittal cross section of airway showing persistent stream of cool air traversing the nasal vestibule and interacting with the CNP. (B) Temperature at the nostril during expiration for the basic airway and the ST airway.

As with *Panoplosaurus*, the basic airway for *Euoplocephalus* consisted of a simple nasal vestibule that extended in a straight line from the nostril to the CNP (Fig 10). The total length of this simplified nasal vestibule was 162.14 mm, which was an 80% reduction in length from the original 808.74 mm nasal vestibule. As with the basic *Panoplosaurus* airway model, we ran this model under our most conservative, Reversed-Reynolds flow estimate. Under this low flow condition, the basic airway of *Euoplocephalus* warmed inspired air by 15.3°C. This initially appeared impressive. However, as with our *Panoplosaurus* model, closer examination of the nasal passage revealed distinct differences between this basic airway and the ST airway. The basic airway of *Euoplocephalus* had a fairly ineffective nasal vestibule. The nasal vestibule provided only 45% of the heat to the air field, resulting in a steady stream of cool air moving through the nasal vestibule and into the CNP (Fig 10). Air field relative humidity still reached saturation, but only after passing into the CNP. Similar to the basic airway of *Panoplosaurus*, this reliance on the CNP to deliver heat to the inspired air field had direct consequences for the nasal passage during expiration, where the nasal passage was only capable of reducing airway temperature by 4.7°C. The resulting expired air left the nasal passage at just 5°C below body temperature (30.3°C).

#### Straightened airway (Fig 2H)

**Fig 11:**
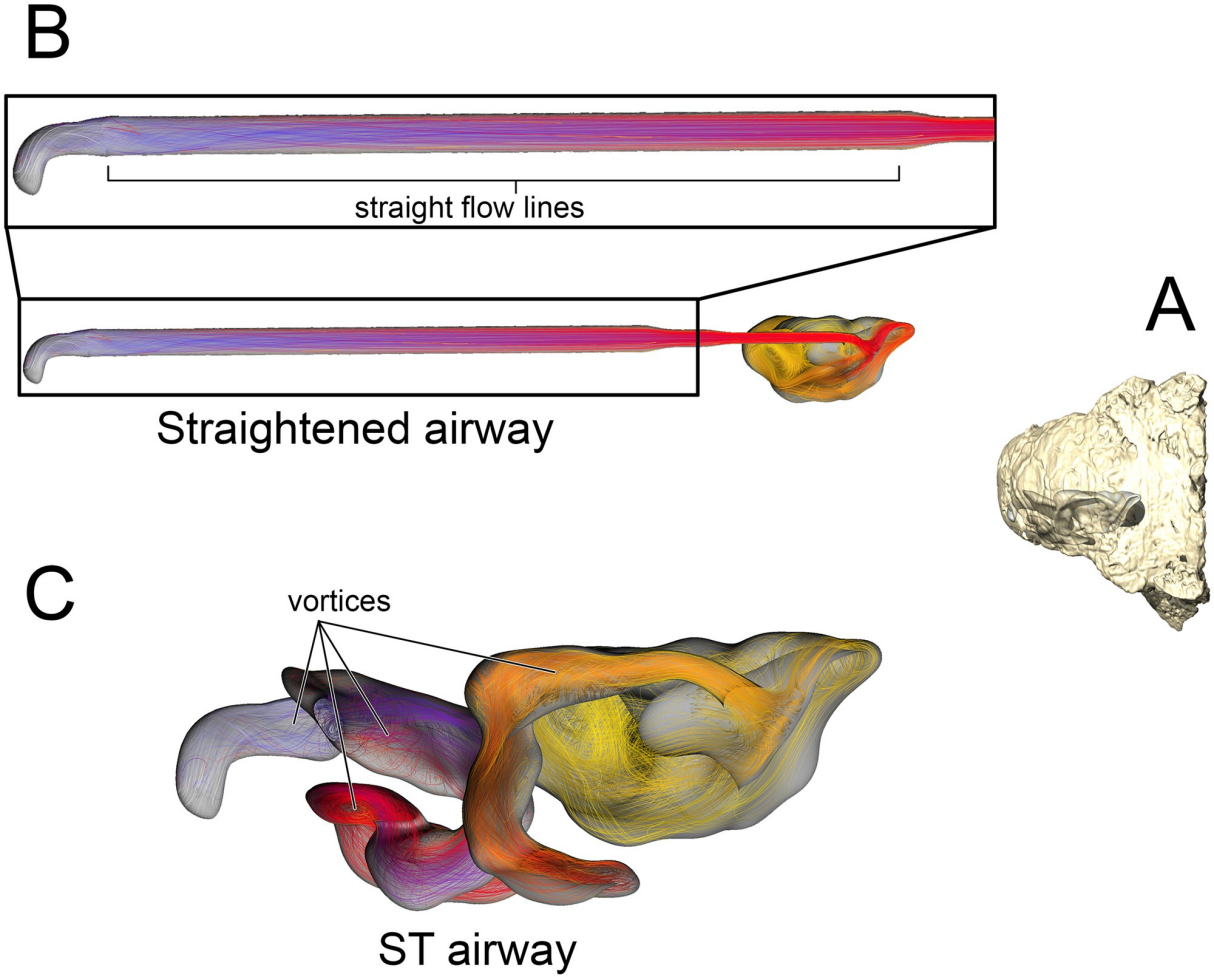
Airflow comparison between the straightened airway and the ST airway in *Euoplocephalus tutus* (AMNH 5405). (A) Dorsal view of the skull of *E*. *tutus* with the left ST airway in situ. (B) Dorsal view of the straightened airway with flow lines in place. Airflow lines are color-coded for temperature (hotter colors = hotter temperatures). *Inset*: Magnified region of nasal vestibule showing evenly spaced, straight flow lines. (C) Dorsal view of the ST airway under the low flow scenario showing the presence of vorticity throughout the nasal vestibule. Note: ST airway in C is not to scale with straightened airway B.

The removal of nasal vestibule curvature resulted in vortex-free, laminar air traversing the nasal vestibule (Fig 11). Under the low flow condition, the straightened airway of *Euoplocephalus* was able to increase the temperature of inspired air by 19.3°C, with 89% of that heating occurring in the nasal vestibule. Water saturation of the inspired air occurred well within the nasal vestibule. On expiration, the straightened airway reduced the temperature of the expired air by 12.2°C, resulting in air leaving the nostrils at 22.8°C.

### Energetic costs vs. savings of air conditioning

The estimated volumes of air that would have been inspired during one breath for *Panoplosaurus* and *Euoplocephalus* were 34 and 64 liters, respectively (Table 2). The energetic cost of heating these volumes of air was 833 thermal calories for *Panoplosaurus* and 1568 thermal calories for *Euoplocephalus*. Calculated energy savings for *Panoplosaurus* and *Euoplocephalus* based on the expired air temperatures are presented in Tables 3 and 4.

**Table 2:** Energetic cost of heating one bolus of air by 20°C at 50% relative humidity for *Panoplosaurus mirus* and *Euoplocephalus tutus*.

Taxon	Tidal volume (ml)	Mass of air (g)	Cost of heating air 20°C (cal)	Latent heat of evaporation (cal)	Total energy cost (cal)
*P*. *mirus*	34000	39	187	646	833
*E*. *tutus*	64000	73	350	1218	1568

**Table 3:** Energy savings from reducing expired air temperature in all airway models for *Panoplosaurus mirus*.

Model	Expired temp (°C)	Heat savings (cal)	Latent heat of condensation (cal)	Total energy recovered (cal)
BB airway (high)	22.67	115	392	507
BB airway (low)	20.46	136	441	577
ST airway (high)	21.57	126	419	545
ST airway (low)	19.48	145	460	605
Basic airway	26.5	79.5	294	374
Straightened airway	23.93	103	363	466

**Table 4:** Energy savings from reducing expired air temperature in all airway models for *Euoplocephalus tutus*.

Model	Expired temp (°C)	Heat savings (cal)	Latent heat of condensation (cal)	Total energy recovered (cal)
BB airway (high)	21.11	244	803	1047
BB airway (low)	19.42	274	868	1142
ST airway (high)	17.34	311	934	1245
ST airway (low)	15.87	337	980	1317
Basic airway	30.3	83	335	418
Straightened airway	22.77	214	738	952

## Discussion

### Critique of methods

#### Nostril placement

The lack of soft-tissue preservation around the nostril makes it difficult to determine just how large the nostril would have been in life, as well as its orientation (lateral vs. terminal). The shape of the nostril has been implicated in directing the air field to parts of the nose in rats and dogs [47,48]. Sauropsids show less nostril mobility than mammals, suggesting that nostril shape is less important for sauropsid nasal airflow dynamics. Nonetheless, the lack of information on nostril shape in extinct animals does limit our knowledge of air field shape in this region of the nose (see Methods). Fortunately, prior studies on how nostril shape alters nasal fluid dynamics indicate that the effects of the nostril on the air field are of limited areal extent, with nasal flow patterns remaining unaffected by nostril placement throughout most of the nasal passage [47].

#### Energy calculations based on V_T_

The caloric energy expenditures and savings that were calculated for each ankylosaur are contingent on our estimates of tidal volume respired during one breath. This tidal volume came from the mass-dependent equations of Frappell et al. [49]. However, as we indicated with flow rate (see Methods), the masses of our two ankylosaurs were substantially greater than those for any of the birds in the dataset of Frappell et al. [49]. Furthermore, the body plan of ankylosaurs is vastly different from their avian relatives, which may negate the use of a tidal volume equation based on birds. However, data from Frappell et al. [49] and Farmer [50] indicate that the tidal volume of archosaurs may be conserved. The mass-dependent equations for tidal volume in birds and crocodylians [49,50] differ only in their coefficients, with that difference being a fairly negligible 0.4. If we used the equation for crocodylian tidal volume instead, it would have increased tidal volume by 1.5–3%, resulting in a 1–3% increase in caloric costs. This fairly small increase in caloric costs would not have changed the comparative results between these two taxa, nor their comparisons to extant animals.

#### Body temperature estimates

Our study specimens were both given a core body temperature of 35°C based on an approximate average taken from our survey of extant, large terrestrial tetrapods (Table 5). Although there has been promising work in paleothermometry using clumped isotopes [51,52], this technique has yet to be applied to any ankylosaur taxon. Thus, it is likely that our estimated body temperature for these two dinosaurs is either too high or too low compared to their actual body temperatures. Despite the arbitrariness of our temperature designation, changing the body temperature to something higher or lower would have only affected the steepness of the heat transfer gradient. Our comparative results would remain the same, with *Euoplocephalus* consistently outperforming *Panoplosaurus*, and the nasal passages of both taxa outperforming their simplified and straightened airway morphologies.

**Table 5:** Core body temperatures recorded for a variety of large, terrestrial amniotes.

Taxon	Body Temperature (°C)	Reference
African elephant (*Loxodonta Africana*)	36.2–36.6	[53,54]
Asian elephant (*Elephas maximus*)	35.7–36.8	[55,56]
Black rhinoceros (*Diceros bicornis*)	31.8–41.9	[57,58]
White rhinoceros (*Ceratotherium simum*)	33.6–37.5	[59,60]
Masai giraffe (*Giraffa camelopardalis*)	35.7–39.1	[33,61,62]
Grizzly bear (*Ursus arctos*)	36.5–38.5	[63]
Emu (*Dromaius novaehollandiae*)	37.4–39.2	[64,65]
Ostrich (*Struthio camelus*)	38.0–40.2	[65,66]
Galapagos tortoise (*Chelonoidis nigra*)	28–31	[67]
Komodo dragon (*Varanus komodoensis*)	36–40	[68]

### Heat transfer efficiency in ankylosaur nasal passages

**Fig 12:**
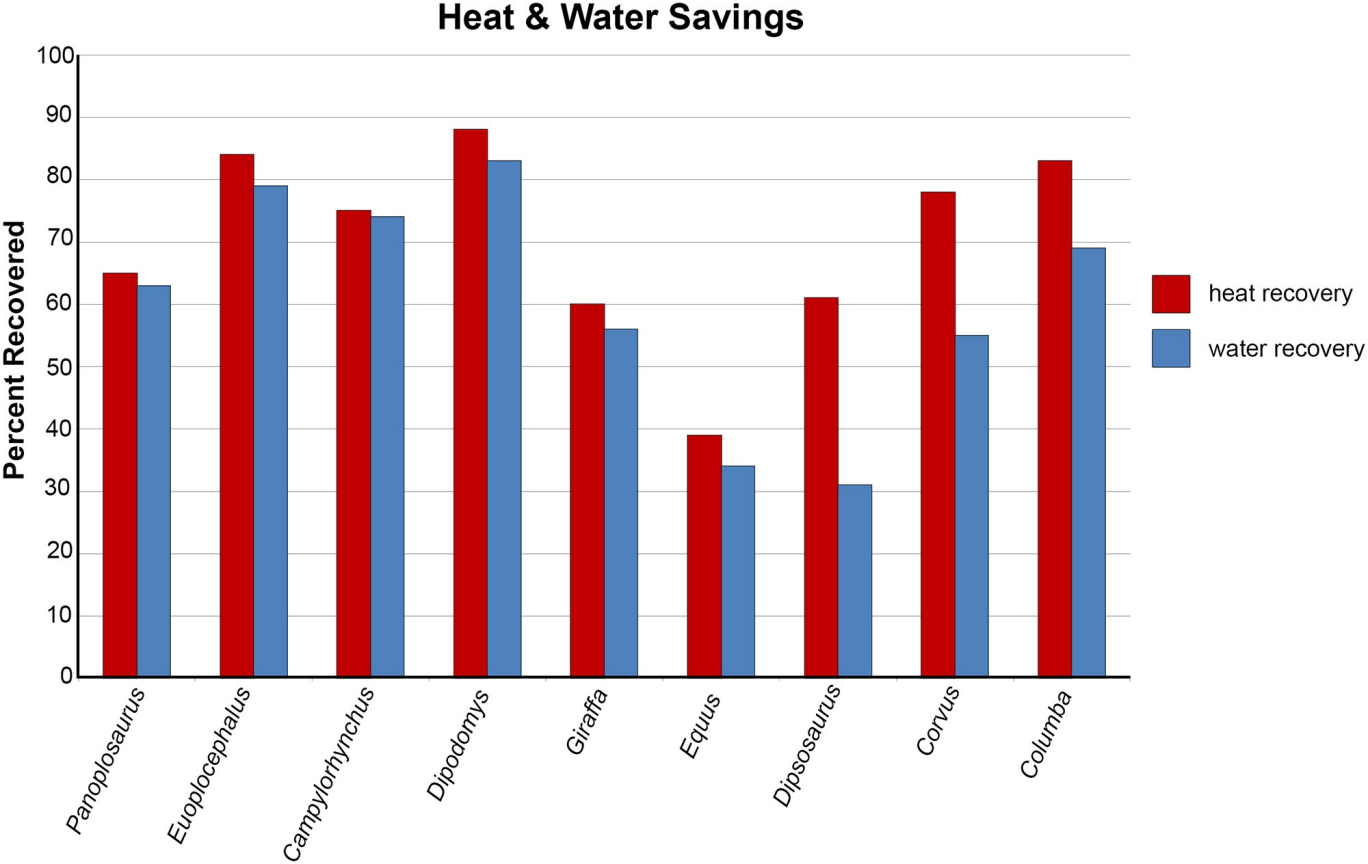
Heat and water savings calculated for the most efficient airway models of *Panoplosaurus mirus* and *Euoplocephalus tutus* vs. various extant animals. Note that variations in experimental protocol means that, although these results are comparable, they should not be viewed as fully equivalent. See Methods for details on graph calculation and references for extant data.

Both ankylosaur nasal passages revealed a substantial capacity to modify the conditions of the air within, indicating that the highly convoluted nasal vestibules of these taxa were efficient heat exchangers. The ST airways under the low flow rate condition, recouped the most energy for both dinosaur taxa (73% and 84% for *Panoplosaurus* and *Euoplocephalus*, respectively). This version of the nasal passage was meant to most closely represent what the original nasal passage would have been like in life. In contrast, the nasal passages as they were preserved in the fossils (i.e., the BB airways) and placed under the same low flow rate conditions, were not able to recoup as much heat energy (69% and 73% of inspiratory cost for *Panoplosaurus* and *Euoplocephalus*, respectively). Despite the remarkably well-preserved nasal passages of both dinosaurs, accounting for soft tissue still resulted in noticeable differences in heat transfer efficiency. Comparing the energy savings calculated for the ST airways of *Panoplosaurus* and *Euoplocephalus* (Fig 2B and 2D) to experimentally obtained energy and water recovery values for extant amniotes, we found both dinosaurs had energy and water recovery values that were on par with many extant animals (Fig 12).

**Fig 13:**
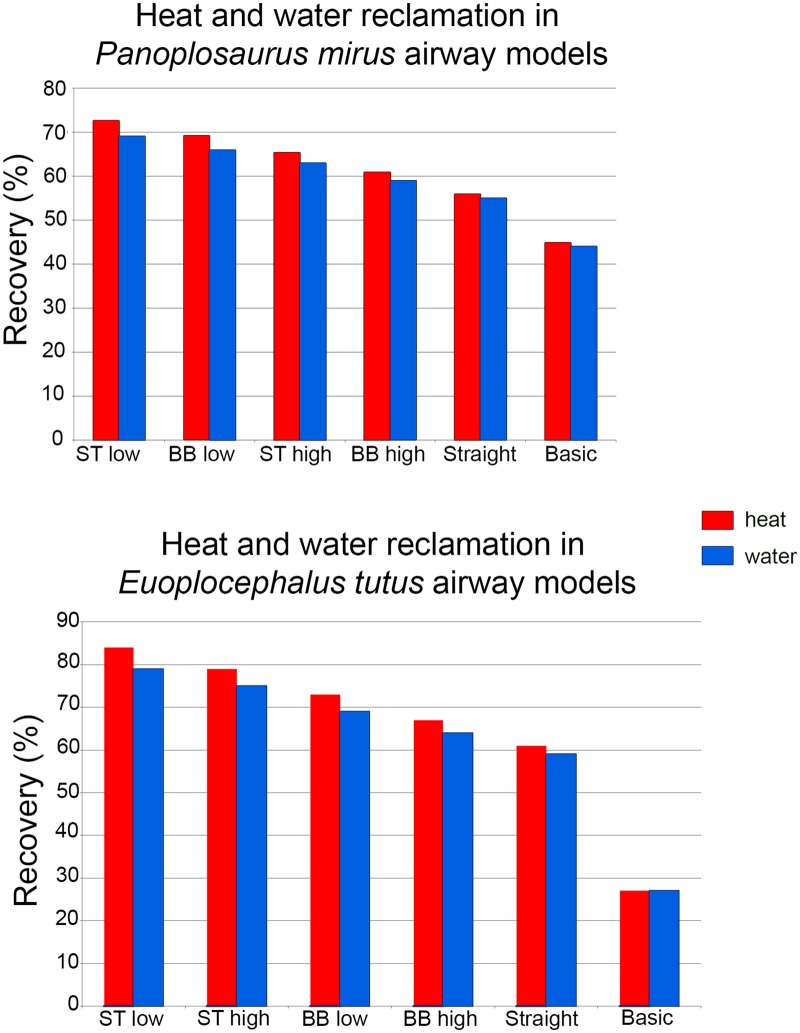
Heat and water savings between all nasal airway models for *Panoplosaurus mirus* (top) and *Euoplocephalus tutus* (bottom). Models are organized from greatest savings to least in both graphs. Abbreviations: ST low, soft-tissue low flow rate; ST high, soft-tissue high flow rate; BB low, bony-bounded low flow rate; BB high, bony-bounded high flow rate; Straight, straightened airway; basic, basic airway.

As predicted, airflow rate did have a noticeable effect on heat transfer efficiency, with lower flow rates resulting in more effective heat transfer (11–14% and 6–9% greater efficiency for *Panoplosaurus* and *Euoplocephalus*, respectively; Fig 13). These results agree with previous measurements and simulations [69,70] that indicate flow rate is one of the most important contributing factors affecting heat transfer between air and the nasal passage.

That the heat transfer efficiency of the dinosaur nasal passages was a result of their great length was made evident upon comparison with the artificially shortened basic airways (Figs 2E, 2G, 6, 10 and 13). These basic airways represented a minimalistic trek of the nasal vestibule from the nostril to the CNP. Achieving this ultra-conservative anatomical shape required excising most of the preserved nasal vestibule (55% and 80% of the nasal vestibule length in *Panoplosaurus* and *Euoplocephalus*, respectively). These truncated nasal vestibules offered a much-reduced surface area for heat and moisture to transfer from the respired air to the nasal mucosa (Figs 6 and 10). Although the CNP did offer a sizeable heat transfer capacity during inspiration, this appears to be due to vorticity within the CNP during inspiration. During expiration, expired air traversed the CNP differently from inspiration. Vorticity was not present and heat transfer through the CNP was minimized, requiring the nasal vestibule to handle the bulk of heat transfer. As such, the basic airways showed extremely reduced heat recovery abilities (62–32% of the respective ST airway heat recovery for *Panoplosaurus* and *Euoplocephalus*, Tables 3 and 4, Fig 13). These results strongly suggest that airway elaboration offers a strong thermoregulatory benefit.

**Fig 14:**
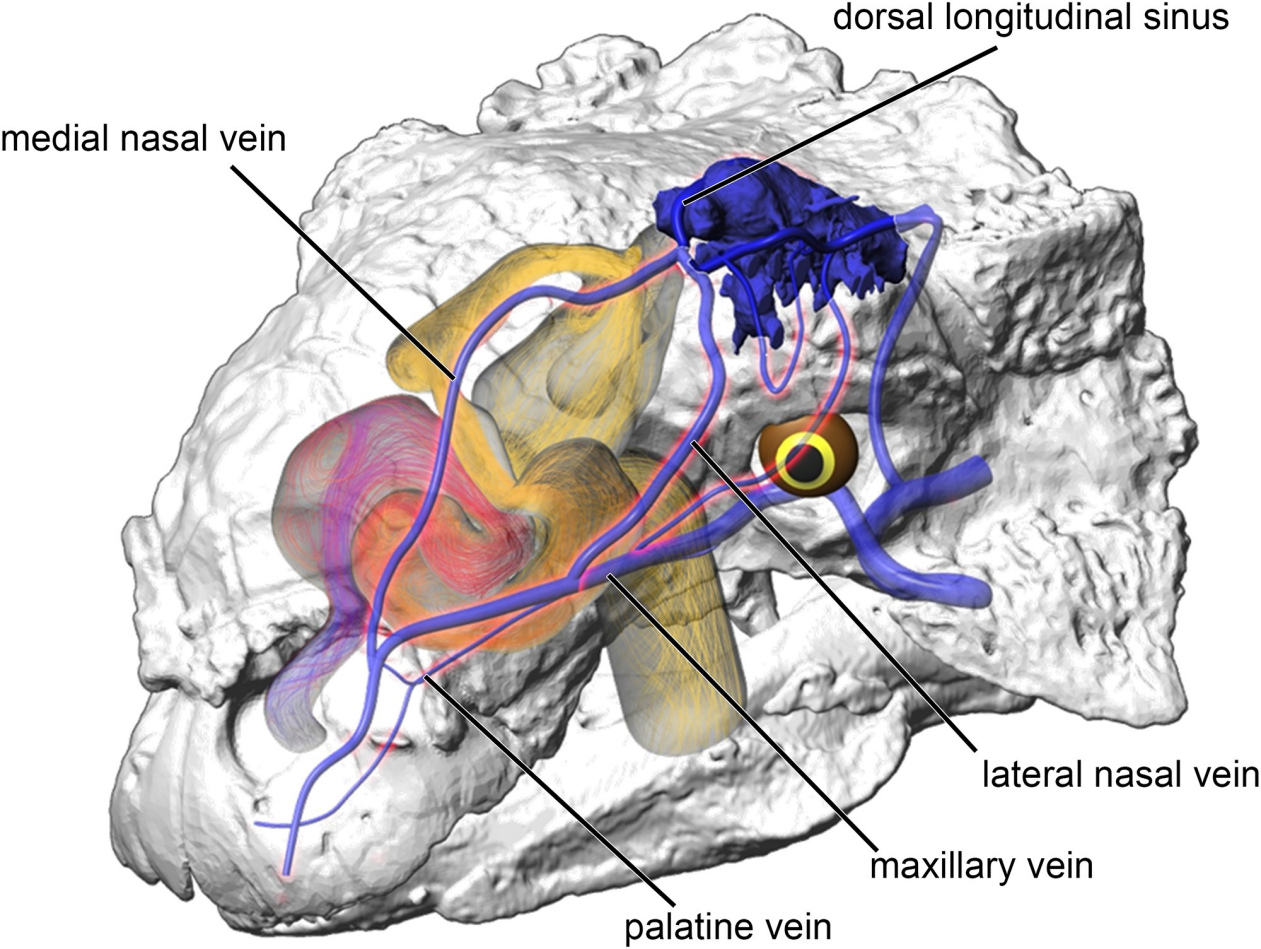
Vascular reconstruction of the venous pathway in the left oronasal apparatus of *Euoplocephalus* (AMNH 5405). Venous reconstruction followed the methods of Porter [72]. Red highlighted veins indicate main channels of heat transfer from the oronasal apparatus to the brain.

Maintaining the length but removing curvature (convolutions) from the nasal vestibule (Fig 2F and 2H) resulted in a negative effect on heat and water recovery ability (Fig 13), albeit not as prominent as the basic airway. When compared to the low flow rate ST airways, curvature removal resulted in a 23% drop in heat transfer efficiency for *Panoplosaurus* and a 28% drop in heat transfer efficiency for *Euoplocephalus*. The removal of airway curvature also removed the presence of standing vortices in the nasal vestibules of both taxa (Figs 7 and 11), which likely explains the reduced heat transfer. As fluid flows within an object (e.g., air in the nasal passage) the portions of the flow field closest to the object’s surface tend to stick to that surface, imparting drag on the fluid as a whole [71]. As streamlines move farther and farther from these surfaces, the sheer imparted by wall drag gets minimized, resulting in fluid at the center of the flow field moving at the highest velocities and producing the classic fluid dynamic parabolic profile [71]. Since fluids at the fluid-surface boundary are essentially static, they create a boundary layer that acts as a barrier to diffusion. For laminar flowing fluids, this boundary layer can be fairly thick. Thus, effective heat transfer through laminar fluids requires a reduction in this boundary layer size [71]. One way to reduce this boundary layer effect is by placing sharp turns and contortions within the nasal passage to break up the boundary layer, allowing cooler air to come into closer contact with the surrounding mucosa. The presence of standing vortices at multiple curves within the nasal passage of both *Panoplosaurus* and *Euoplocephalus* (Figs 7C and 11C) revealed multiple regions where that boundary layer was broken up. Further, the presence of vortices acts to slow down the passage of the air molecules through the nasal passage, providing more time for air to reach thermal equilibrium with the body. By coiling the nasal passage within the skull, ankylosaurs were able to take advantage of the extra surface area for air to interact with the mucosa. This surface area, coupled with the adjacent location of large nasal vasculature ([20,72] Fig 14) and boundary-layer-breaking effects produced by forcing the air field to radically alter direction as it moved through the nasal vestibule, resulted in these nasal passages acting as very effective air conditioners.

The ST airway reconstructions (Fig 2B and 2D), under the low flow rate conditions, produced the highest water savings (69% and 79% for *Panoplosaurus* and *Euoplocephalus*), whereas the truncated, basic airways were the least effective (44% and 27%) at water reclamation. Our data suggest that the nasal passages in both ankylosaurs could have functioned effectively as water reclaimers as well as air conditioners.

### Heat transfer in *Panoplosaurus* vs. *Euoplocephalus*

**Fig 15:**
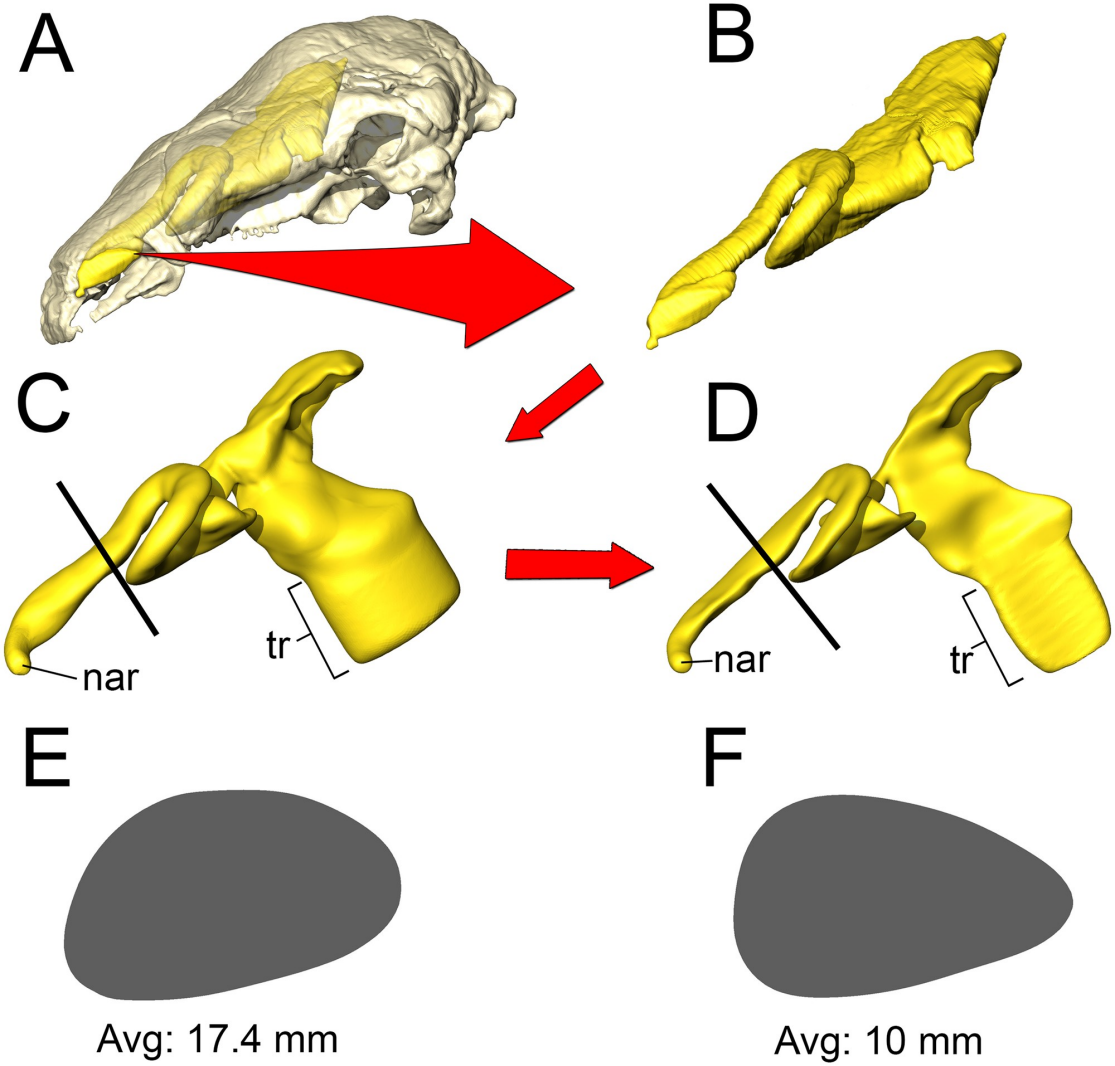
Airway reconstruction and soft-tissue correction in *Panoplosaurus mirus* (ROM 1215). (A) Initial CT-based bony-bounded segmentation of airway within the skull and (B) isolated BB airway. (C) Airway cleaned and separated, with the addition of a soft-tissue naris and nasopharyngeal duct exiting into an artificially created laryngotracheal region. (D) Nasal passage digitally compressed to reduce airway caliber, better simulating the mucosa-lined airways of extant amniotes. Black lines indicate locations of cross sections (E–F). (E) Cross section of original BB airway caliber. (F) Cross section of airway after soft-tissue correction.

**Fig 16:**
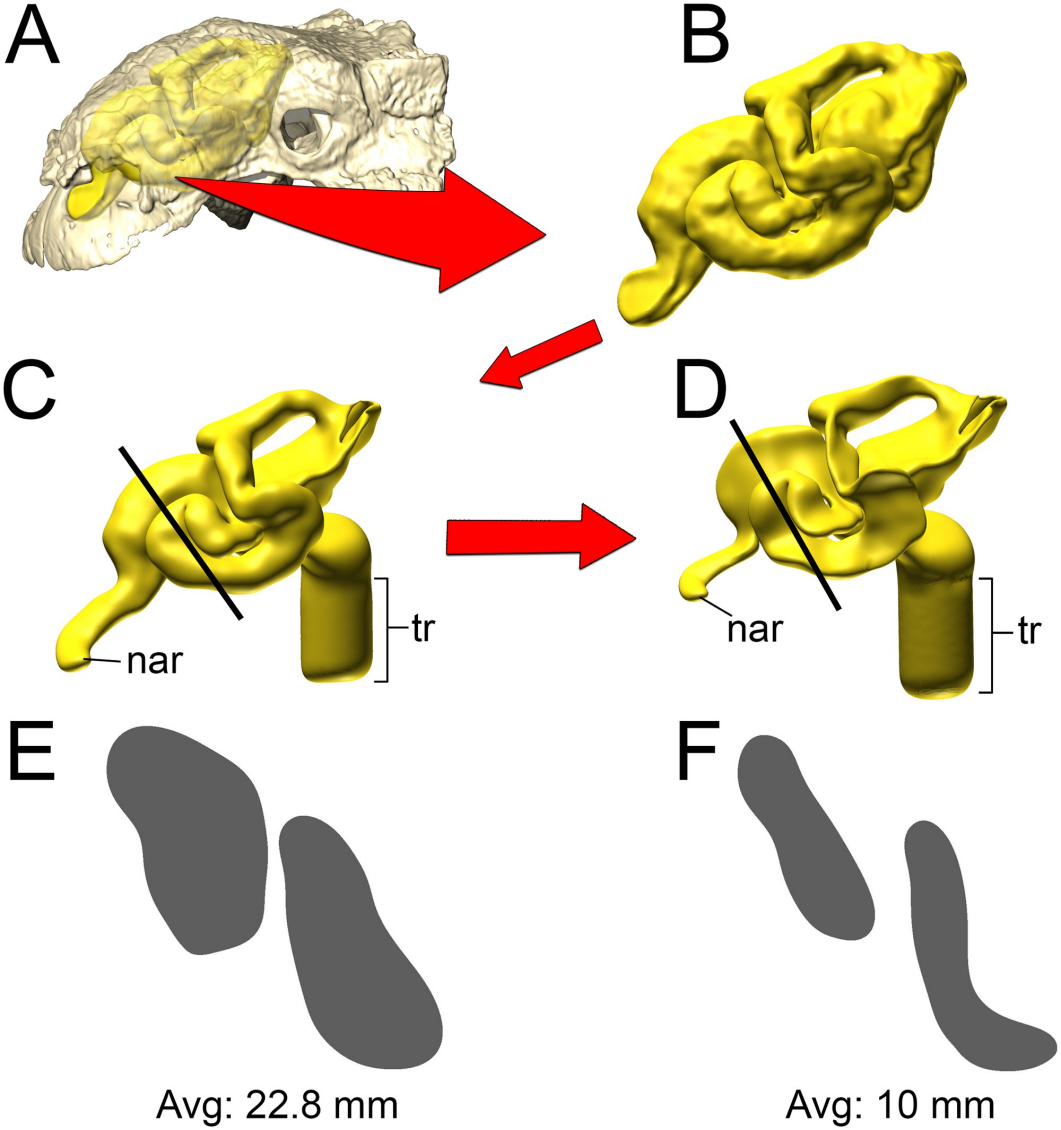
Airway reconstruction and soft-tissue correction in *Euoplocephalus tutus* (AMNH 5405). (A) Initial CT-based bony-bounded segmentation of airway within the skull and (B) isolated. (C) Airway cleaned and separated, with the addition of a soft-tissue naris and nasopharyngeal duct exiting into an artificially created laryngotracheal region. (D) Nasal passage digitally compressed to reduce airway caliber, better simulating the mucosa-lined airways of extant amniotes. Black lines indicate locations of cross sections (E–F). (E) Original bony-bounded airway caliber. (F) Airway caliber after soft-tissue correction.

**Fig 17:**
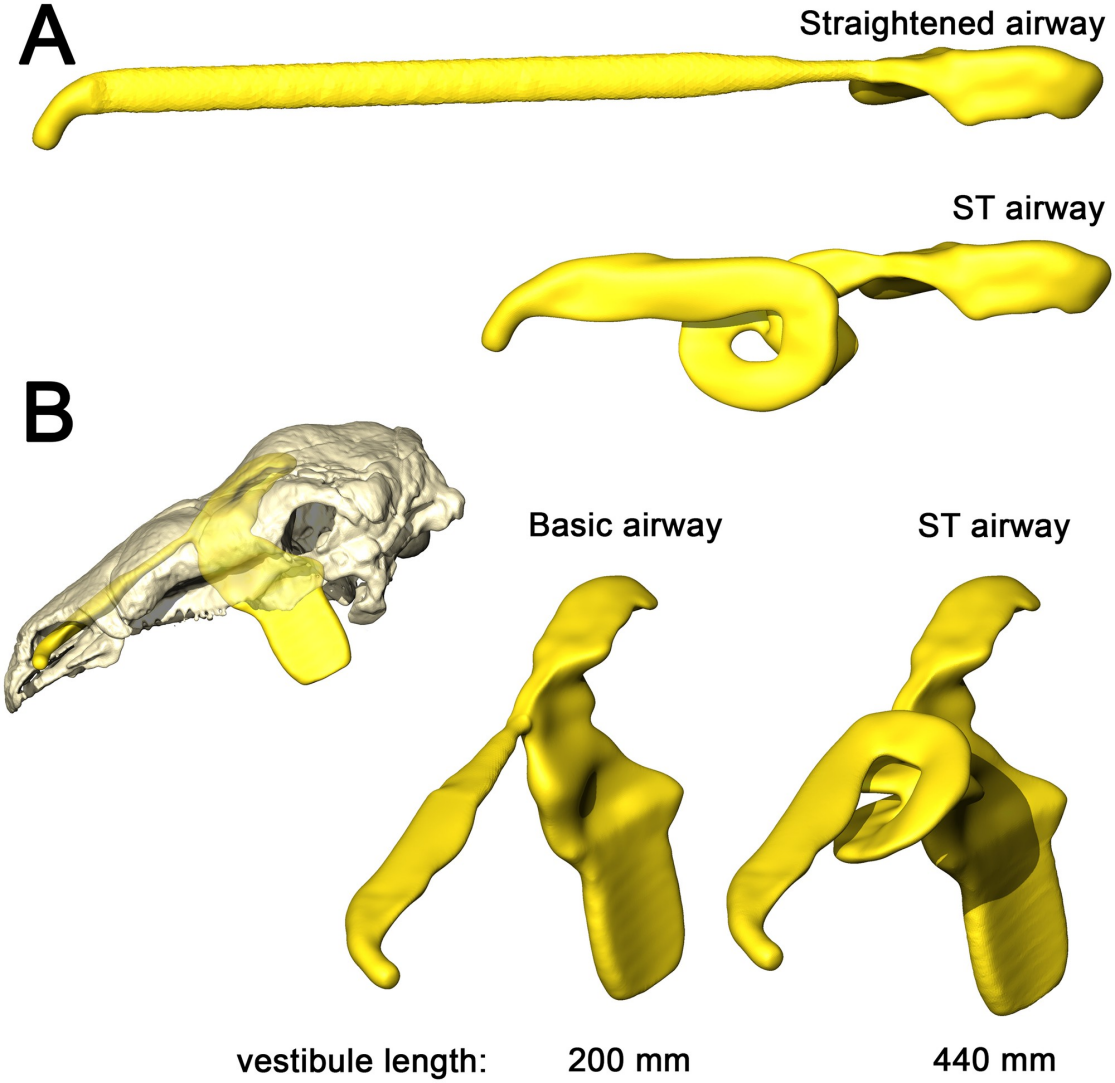
Alternate airway models for *Panoplosaurus mirus*. (A) Dorsal view of the straightened airway (removal of nasal vestibule curvature) and the original, ST-corrected airway. (B) Lateral view of skull of *P*. *mirus* (ROM 1215) with basic airway in situ. A direct connection between the bony narial aperture and the CNP in a loss of 55% of the original nasal vestibule.

**Fig 18:**
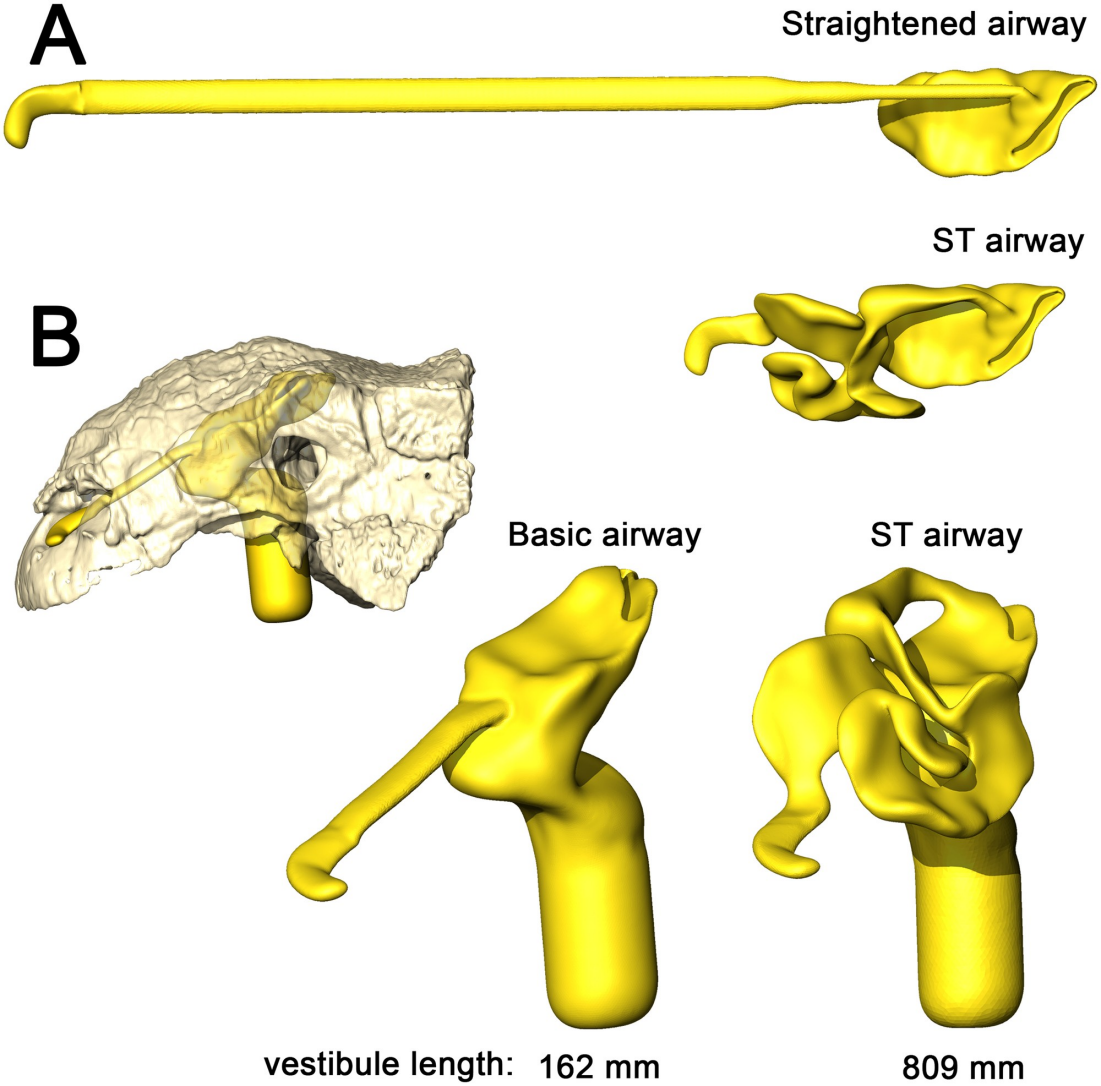
Alternate airway models for *Euoplocephalus tutus*. (A) Dorsal view of the straightened airway (removal of nasal vestibule curvature) and the original, ST-corrected airway. (B) Lateral view of skull of *E*. *tutus* (AMNH 5405) with basic airway in situ. A direct connection between the bony narial aperture and the CNP resulted in a loss of 80% of the original nasal vestibule.

A consistent trend observed throughout this study was the greater heat transfer efficiency in the nasal passage of *Euoplocephalus* (Table 4, Fig 13 bottom) as compared to *Panoplosaurus* (Table 3, Fig 13 top). Under both high and low flow rate conditions, both the BB and ST airways of *Euoplocephalus* (Fig 2C and 2D) were able to bring inspired air closer to simulated body temperature than the respective airway models of *Panoplosaurus* (Fig 2A and 2B). Upon expiration, the ST nasal passage of *Euoplocephalus* was able to lower air temperatures 3.6–4.2°C lower than air temperatures in the ST-corrected nasal passage of *Panoplosaurus* (Figs 4, 5, 8 and 9). This translated into a 15–22% greater energy savings and a 14–19% greater water recovery. The more elaborately convoluted nasal vestibule in *Euoplocephalus* compared to *Panoplosaurus* ([20], Figs 15–18) was likely responsible for these greater energy savings despite the larger flow rates and tidal volumes. The nasal vestibule played the largest role in air conditioning for both models. These results indicate that *Euoplocephalus* had a more effective nasal passage than *Panoplosaurus* in relation to heat transfer. The evolutionary pressures behind these different performances are difficult to decipher.

Both ankylosaurs are known from the same stratigraphic level of the Dinosaur Park Formation [73], indicating that they were sympatric. As such, it is unlikely that gross environmental factors were responsible for the more elaborate nasal passages of *Euoplocephalus*. It is possible that the more elaborate nasal passage in *Euoplocephalus* was simply a byproduct of ankylosaurian phylogeny and ecology. *Euoplocephalus* and *Panoplosaurus* represent the two major divisions of the clade Ankylosauria [1, 58]. Currently, our knowledge of nasal passage shape in ankylosaurs is limited to these two taxa. However, if the more extensive “paranasal sinus system” of ankylosaurids [16,18] is an indication of a more convoluted nasal vestibule, then ankylosaurids would have apomorphically elaborated their nasal passages to a greater degree than nodosaurids. The driving force behind the more extensive elaboration of the nasal vestibule in ankylosaurids over nodosaurids is difficult to determine. The notably divergent cranial architecture in these two taxa likely played an important part. Nodosaurid skulls are longer than they are wide, appearing pyriform in dorsal view, whereas ankylosaurid crania are decidedly squatter with broad and blunt rostra [12]. The more restricted cranial real estate in ankylosaurids would necessitate greater convolutions to obtain an equivalent airway length to nodosaurids. With that said, it is pertinent to note that the length of the nasal vestibule in *Euoplocephalus* greatly exceeds that observed in *Panoplosaurus*, regardless of convolutions (Table 6). Based on our mass estimates of these two taxa, *Euoplocephalus* was 1.7–1.8 times larger than *Panoplosaurus*. However, the cavity housing the nasal vestibule of *Euoplocephalus*—as preserved in the cranium—was just over twice the length and nearly 3 times the volume of the preserved nasal vestibule cavity in *Panoplosaurus*. Skull architecture does not appear to fully explain the discrepancy in nasal passage length between these two taxa.

**Table 6:** Nasal vestibule size compared with body mass and endocast volume.

Taxon	Vestibule length (mm)	Vestibule volume (mm^3^)	Body mass (kg)	Endocast volume* (mm^3^)
*Panoplosaurus*	400	157134	1100–2000	699.5
*Euoplocephalus*	808.74	436740	2000–3500	811.5

*Endocast volume obtained from segmentations of endocranial cavity by Witmer & Ridgely [20,21]

Cranial differences between nodosaurids and ankylosaurids have been argued to be a response to the different dietary niches of these two ankylosaur clades. The narrower snouts of nodosaurids suggest a more selective feeding strategy, as compared to the bulk feeding proposed for ankylosaurids [74]. The Dinosaur Park Formation has been interpreted as housing both open and closed habitats [74]. If ankylosaurids were less discerning in their diets and relied heavily on hindgut fermentation, they could have spent more time grazing in open terrain. This extensive time under a constantly-beating sun, coupled with the heat produced from vast quantities of fermenting vegetation in the gut, may have placed a higher heat load on the brain of *Euoplocephalus*, as opposed to *Panoplosaurus*, which could have spent more time in covered habitat, with less reliance on hindgut fermentation of low-quality ingesta. Although this scenario is largely speculative, it would align with previous work on dental microwear and expected diets in ankylosaurids as compared to nodosaurids [74,75].

A final factor to consider is the overall size difference between these two ankylosaurs. *Euoplocephalus* was 900–1500 kg more massive than *Panoplosaurus* based on estimates from Brown et al. [73]. Larger animals—with their lower surface-area-to-volume ratios—absorb, produce, and retain more heat than smaller animals [76]. As a substantially more massive animal, *Euoplocephalus* would have been capable of absorbing higher heat loads than its lighter relative. Elaboration of the nasal apparatus could have been an evolutionary response to offsetting these larger heat loads. The Dinosaur Park Formation featured two other ankylosaurs during this time period. The nodosaurid, *Edmontonia rugosidens* and the ankylosaurid *Dyoplosaurus acutosquameus*. Both taxa are known from enough material to provide fairly accurate estimates of their body mass [73]. Notably, both ankylosaurids had higher estimated body masses for a respective body length than their nodosaurid relatives (Table 7). Ankylosaurids appear to have been heavier nodosaurids for any given body length.

**Table 7:** Size estimates of ankylosaurs from the Dinosaur Park Formation.

Taxon	Estimated length (m)	Estimated mass (kg)*
*Dyoplosaurus*	3.58–4.16† [77]	1500–2500
*Euoplocephalus*	5–6† [77]	2000–3500
*Edmontonia*	6‡ [78]	1000–1800
*Panoplosaurus*	5‡ [78]	1100–2000

* Brown et al. 2013 [73]

^†^ Arbour et al. 2017

^‡^ Paul 2016

The potentially more massive bodies in ankylosaurids suggests that a potential causal relationship between nasal passage complexity and body size may exist. This relationship aligns well with the heat transfer results from our simulation study. Further supporting this causal relationship was a recent CT analysis of the small, basal ankylosaur *Kunbarrasaurus ieversi* [79]. The authors discovered its nasal passage to be remarkably short and potentially simplistic, which suggests that ankylosaur nasal passages became more elaborate as members of the clade grew larger. However, *Kunbarrasaurus* did also live in a different environment and time from the ankylosaurs in our current study, and thus was likely subjected to a different set of environmental pressures. Detangling nasal passage shape from the multiple factors of ecology, phylogeny, and biology is a topic in of itself that is beyond the scope of this study. It is likely that a mix of all three of these factors drove the evolution of nasal passage elaboration in ankylosaurs. Further, these results do not negate potential alternate functions of the nasal passage, especially in regard to vocal resonance. Our results only indicate that the elaborate nasal passages of ankylosaurs had the potential to be efficient heat exchangers, even if that was not their primary function.

### Nasal convolutions vs. respiratory turbinates

Regardless of the relative efficiency between these two ankylosaurs, both animals seemed quite capable of modifying respired air. That both dinosaurs were able to modify respired air without the aid of respiratory turbinates or conchae is particularly intriguing. Respiratory turbinates—and the physiologically active mucosal conchae that reside on them—have been hypothesized to have evolved strictly for the function of increasing the water reclaiming ability of the nasal passage, mitigating the effects of high ventilation rates as seen in mammalian endotherms [34,35,38,39]. However, as has been previously suggested [80,81], this necessity for respiratory turbinates appears to be truer for mammals than for sauropsids, as the latter have markedly lower ventilation rates than equivalently sized mammals [49,80]. The estimated resting breathing frequency of *Panoplosaurus* and *Euoplocephalus* was 1.5 and 1.2 breaths/minute, respectively. Such slow breathing would result in naturally low rates of respiratory evaporative water loss (REWL) regardless of whether respiratory turbinates were present. As such, the need for a water recovery mechanism may not have been as strong a selective force as it appears to be in mammals, allowing for alternate means of solving the REWL problem, such as evolving a long, winding nasal passage. Our results indicate that an elongated, convoluted nasal passage produces equivalent results to a turbinate-filled airway. Both anatomical organizations appear to offer the same results albeit with different tradeoffs. A single, winding airway warms and transports air in a stepwise, serial fashion. In contrast, a turbinate-filled airway breaks the air field into multiple, parallel-running air streams. The latter approach appears to function well at warming large volumes of air in a relatively small space, allowing a short airway to act like a long airway [82]. However, by breaking the airfield into a series of smaller streams, turbinates also decrease the caliber of the airway in these regions. Intuitively, we should expect to see a concomitant, and rather large, increase in airflow resistance as determined from a derivation of the Hagen-Poiseuille equation [71]:

$$R=\frac{8\mu l}{\pi r^{4}}$$ (1)

where R = resistance, μ = dynamic viscosity, l = the length of the “pipe” and r = the radius of the “pipe.” As indicated by the exponent in the equation, resistance is highly sensitive to the radius of the structure through which a fluid flows. Separating the airfield into a series of smaller air channels should result in a substantial increase in airway resistance. However, even though turbinates break up the airway into multiple channels, these channels are all running in parallel to each other. Unlike resistance in a serial system of pipes, the cumulative resistance in a turbinate-filled airway—much like in an electrical circuit—is best calculated by taking the reciprocals of resistance for each parallel channel [71]:

$$R_{total}=\sum \frac{1}{\frac{1}{R_{1}}+\frac{1}{R_{2}}+\frac{1}{R_{3}}\ldots }$$ (2)

**Fig 19:**
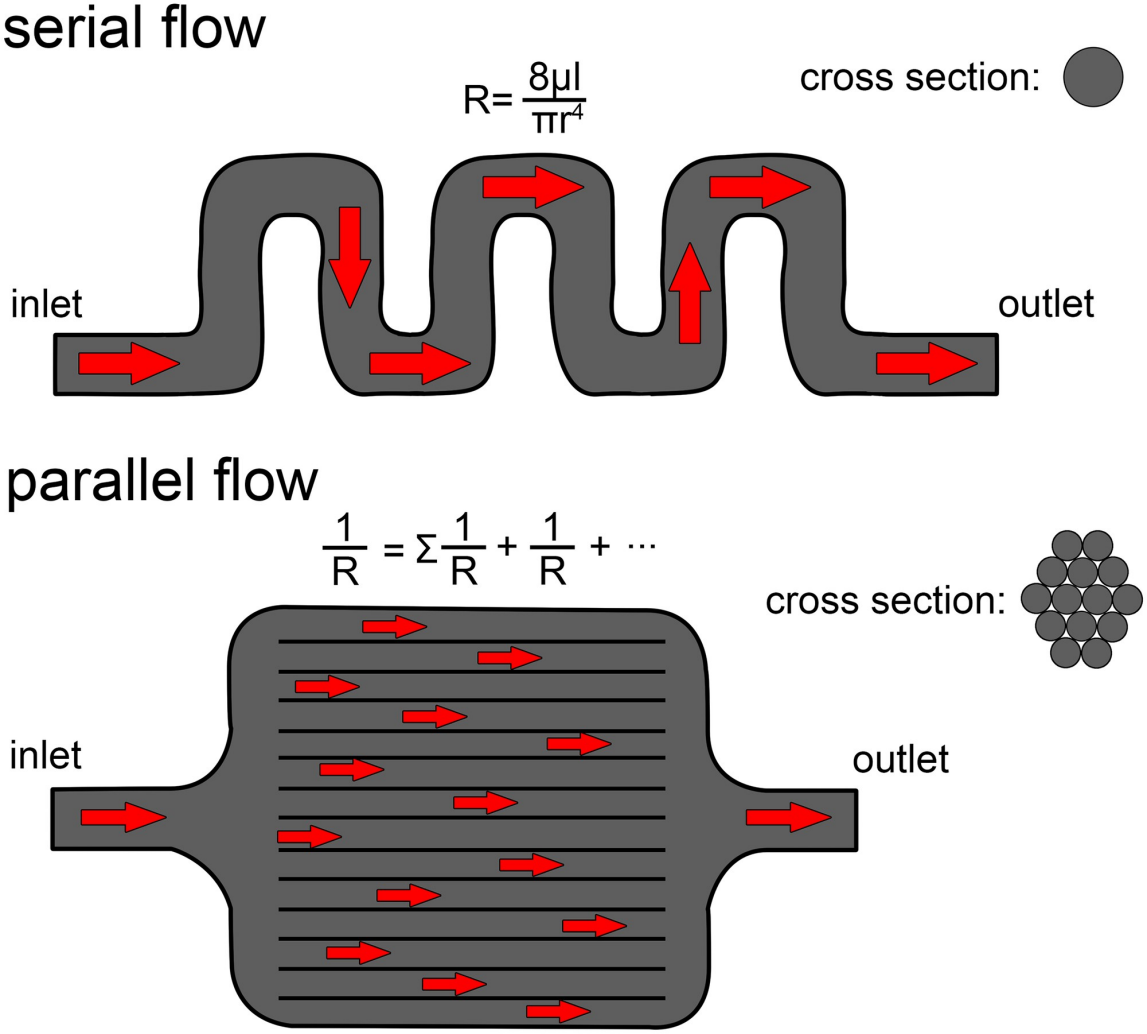
Example of flow in a hypothetical, serial pipe (top) vs. a parallel pipe (bottom). Resistance is sensitive to the pipe’s caliber, giving the parallel pipe greater resistance on the outset. However, the parallel arrangement of the smaller caliber tubes offsets some of the increased resistance, resulting in only a modest increase in overall resistance, while simultaneously increasing surface area to volume ratios. In the above example, the parallel pipe has half the caliber of the serial pipe, but is split into fourteen partitions, resulting in approximately identical resistance to the single, serial pipe.

**Fig 20:**
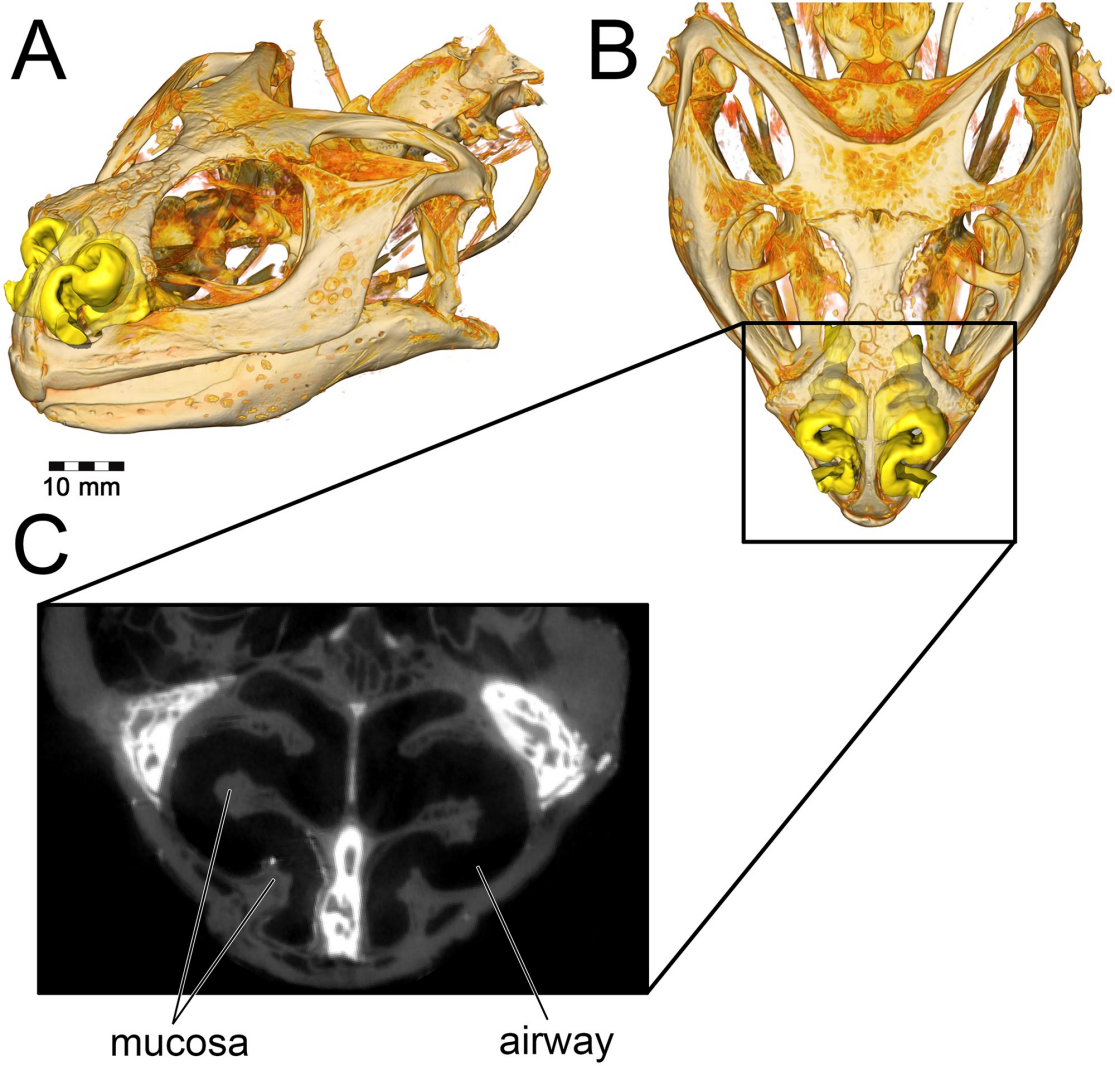
**Airway of the lizard *Uromastyx aegyptia* (OUVC 10688) in (A) oblique left lateral and (B) dorsal view**. As with ankylosaurs, the nasal passage (yellow) exhibits convolutions that increase surface area. (C) Horizontal CT slice image reveals that “slabs” of mucosa are responsible for compressing the airway. It is likely that these mucosal slabs are well vascularized, which would aid in heat and water savings during respiration in this taxon.

Thus, an airway split into multiple parallel channels will increase in resistance much slower than it would appear at the outset, which makes the filling of nasal passages with turbinates an energetically viable option (Fig 19). Standard scaling rules indicate that an isometric increase in body size will increase the volume of the nasal passage [76]. Van Valkenburgh et al. [83] found the turbinate-filled nasal passages of carnivorans to scale positively allometrically, thus indicating even larger increases in airway volume with body size than predicted from isometry. Increasing nasal passage volume should reduce the efficacy of the nasal passage at transferring heat, due to the diffusion limited nature of heat transfer discussed earlier. However, increasing the volume of a turbinate-filled nasal passage results in a relatively minor increase in the gap distance between opposing turbinate walls. Thus, an increase in nasal cavity volume should result in only modest increases in individual channel volume. The larger size of the nasal cavity may further be compensated for by changing the arrangement of individual turbinates (e.g., increased scrolling) or increasing the thickness of the mucosa that resides on them (conchae). This ability to slow the rate of radial distance increase within the air field appears to make nasal turbinates more resistant to changes in body size than a serially-arranged nasal passage may be. This resilience may explain why turbinate density appears to not scale with body size in carnivoran mammals [83]. In contrast, a single long airway offers lower resistance, but it does impose a limit on how much air can be processed at any one time. Increasing heat-transfer efficiency—or maintaining it at larger sizes—requires more contortions of the nasal passage, filling up more space within the skull and increasing anatomical dead space within the conducting portion of the respiratory system. For slow-breathing animals this limitation on airflow processing is less of a problem. Among extant diapsids, the nasal passages of ankylosaurs are reminiscent of the winding airways found in some lizards [84], such as *Uromastyx* (Fig 20). Lizards—much like birds—are slow breathers compared to similar sized mammals [85], and the limitation on air processing imposed by a long, serial airway does not seem to affect them. Further the likely presence of a unidirectional airflow system in the lungs of dinosaurs [86,87], and possibly all diapsids [88–90], would further offset any increases in anatomical dead space.

### Nasal air conditioning and brain cooling

Although much has been written on the heat and water retaining function of respiratory turbinates [34–39] as well as the nasal passage itself [28,29,31–33], fewer studies have looked at the other side of this nasal function, namely, its ability to dump excess heat from the body core. Inspired air is heated in the nasal passage by pulling heat away from warm blood coursing beneath the adjacent mucosa. As such, the nasal passage not only warms and humidifies inspired air, but it also acts as a heat sink for hot blood coming from the body core. Blood vessels that surround the nasal passage have the potential to shed excess heat, resulting in a source of cooled blood. An extensive survey of vasculature in extant sauropsids [72,91–93] found that the nasal passage is supplied by predictable branches from the internal and external carotid arteries. These arterial branches supply capillary beds within the nasal mucosa that transition into major venous pathways that course caudally towards the brain and eyes. Reconstructed vasculature in dinosaurs, including the ankylosaurs used in this study ([20], Fig 14), has revealed extensive nasal vasculature in large-bodied dinosaurs with pathways similar to those of extant diapsids [72,80,91]. Shedding excess body heat in the nasal passages provides a means for sauropsids to keep their brains from overheating and maintain an independent and physiologically optimal temperature range. Studies on extant amniotes have found that head temperature tends to be more strictly regulated than body temperature [94–98]. The oronasal apparatus has long been implicated in controlling these body temperature differences [98–102]. Many studies have focused solely on the role of the oronasal apparatus in avoiding heat stress (e.g., [102,103]). These studies tended to observe other signs of heat stress (e.g., panting) that have been shown to offer a means of locally cooling the cephalic region of the body [100–102]. Our ankylosaur data, however, indicate that a substantial blood cooling capacity in the nasal passage was present even under “relaxed” or resting conditions (i.e., not heat stress). We speculate that heat dumping in these enhanced nasal passages may have been more obligate as the large size of these dinosaurs would have resulted in a very high heat load that—when transferred to remarkably small brains ([83], Table 6)—would have created conditions that would continuously place the brain at risk of overheating. Interestingly, similar nasal elaborations have been observed in sauropods, ornithopods, and ceratopsians [104]. All of these groups are comprised of mostly large bodied (multi-tonne) animals. As with ankylosaurs, these dinosaur taxa also reveal elaboration of the nasal vestibule. The nasal vestibule appears to be the most plastic of nasal passage anatomy, as it is also the most variable part of the nose in birds, turtles, and lizards [105–108]. Nasal vestibule elaboration in sauropods, hadrosaurs, and ceratopsians may have evolved for brain cooling in these taxa as well. Future work looking at more basal members of these dinosaur groups (e.g., the basal ankylosaurian, *Kunbarrasaurus* [109]) will provide greater insight into the role of nasal passage elaboration and body size evolution in dinosaurs.

## Materials and methods

### Specimens

We analyzed airflow in two species of ankylosaur, the nodosaurid *Panoplosaurus mirus* (ROM 1215) and the ankylosaurid *Euoplocephalus tutus* (AMNH 5405). CT data and initial 3D models were obtained from previous work conducted by Witmer & Ridgely [20]. To aid with soft-tissue reconstruction, we further looked at other specimens of *Euoplocephalus* (AMNH 5403, ROM 1930) and *Panoplosaurus* (CMN 2759) as well as the related species: *Edmontonia rugosidens* (AMNH 5381), *Ankylosaurus magniventris* (AMNH 5214), *Pinacosaurus grangeri* (ZPAL MgD-II/1), and *Kunbarrasaurus ieversi* (QM F18101).

### Model construction

#### Segmentation

**Fig 21:**
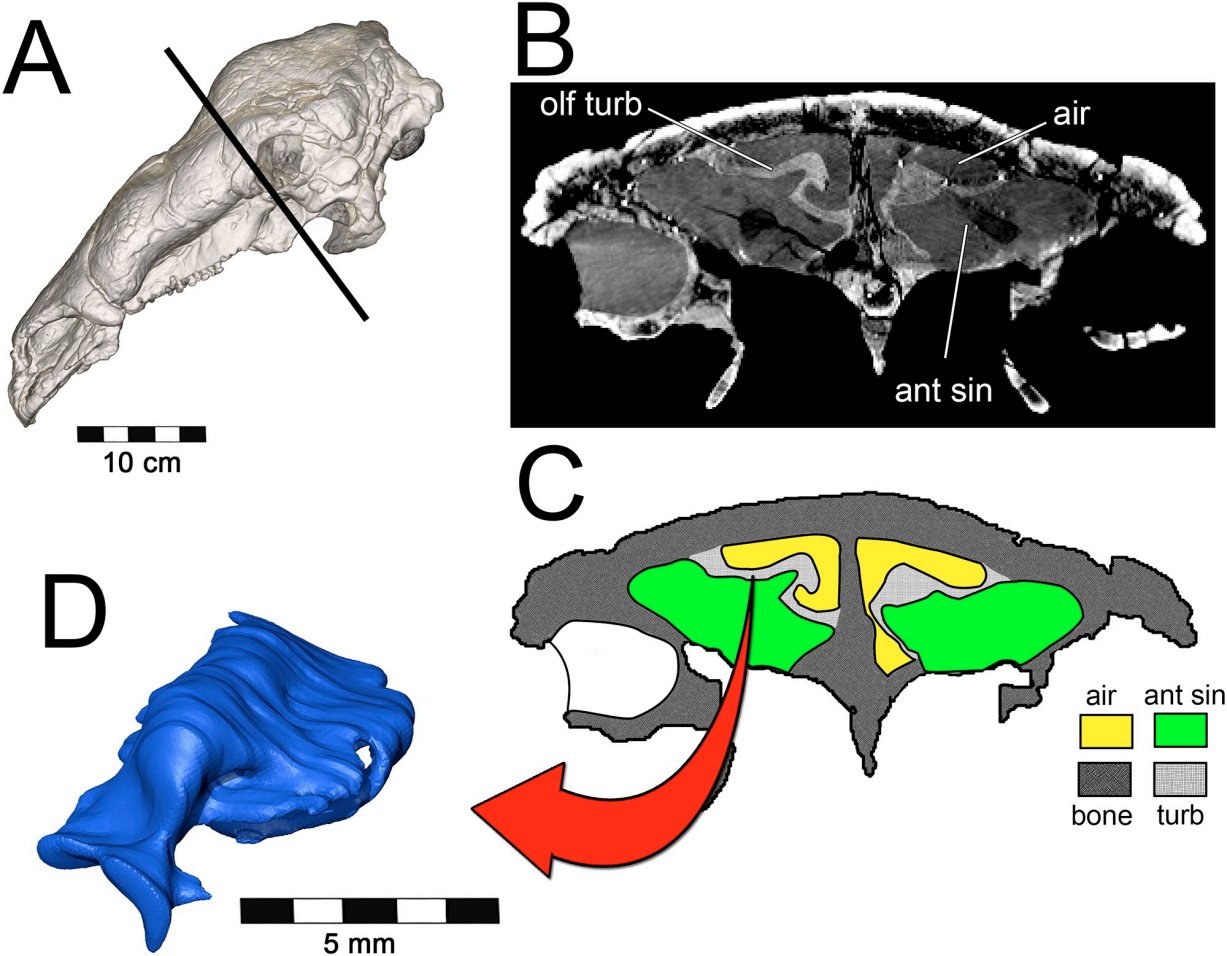
Segmentation of the airway in *Panoplosaurus mirus* (ROM 1215). **(A) Skull in left lateral view**. Line represents the location of (B) axial CT section showing preserved olfactory turbinates. (C) Diagram of CT image showing caliber of airway vs. entire nasal cavity. (D) Segmented olfactory turbinate in same plane as CT image.

Osteological evidence for a complete nasal septum (septal sulcus or partially mineralized septum) meant that left and right nasal passages acted independently of each other. This allowed for modeling of one side of the nose only, which saved on computational costs. Initial segmentation of the airways produced a rough approximation of the nasal passage in life, complete with a rostral and caudal vestibular loop [20] and an enlarged olfactory recess (Fig 2). We refer to the enlarged, looping area of the nasal passages in both ankylosaurs as the nasal vestibule. This demarcation of nasal passage anatomy in sauropsids is typically determined by the placement of the duct for the nasal gland [105,106]. Unfortunately, the duct and its ostium are both soft-tissue structures that do not leave impressions on the bone. As an alternative, we used the region of the nasal passage where nasal cavity diameter suddenly increased [110]. This area is known to correlate with the terminus of the nasal vestibule in extant sauropsids.[111] Further, support for this interpretation comes from the tube-shaped structure of the nasal vestibule in extant reptiles. The morphology of the looping portions of the ankylosaur airways best fits this description. The nasal vestibule and the CNP were unattached to each other in the original airway segmentations [20], requiring further segmentation and attachment to produce a contiguous surface model. The nasopharyngeal duct was not segmented in the original models and required segmentation. Although Witmer and Ridgely [20], as well as Miyashita et al. [21], discussed the presence of well-preserved olfactory turbinates within the olfactory recess of these dinosaurs, neither study published images of the segmented structures. For our study, these structures and their effect on the airway (i.e., the impressions they left on the digital airway cast) were segmented using the program Avizo 7.1 (FEI Visualization Sciences Group, Burlington, MA). As with the initial airway segmentation, the final product was a cast of the inside of the nasal cavity, revealing the potential space in which air could reside within the nasal passage. Examination of the CT data within the olfactory recess revealed both the presence of mineralized olfactory turbinates as well as the outer boundary to the nasal capsule. These data provided insight into the limit of the airway in life, which was substantially more restricted than initial segmentations suggested (Fig 21). Extra space lateral to our interpretations of the nasal passage wall was interpreted as housing the antorbital sinus. Its placement near the olfactory chamber, adjacent to the CNP, is consistent with antorbital sinus placement in extant archosaurs [14]. In preparation for meshing, the segmented models were cleaned of segmentation artifacts using the program Geomagic 10 (3D Systems Geomagic, Rock Hill, SC).

#### Fleshy nostril placement and soft palate

Although ROM 1215 and AMNH 5405 both contained well preserved nasal passages, the terminal regions of the nose—the fleshy nostril and choana—were not preserved. To aid with these soft-tissue reconstructions we turned to other specimens of the same species along with well-preserved specimens of related ankylosaurs to better determine the location of the nostril and choana.

#### Fleshy nostril

**Fig 22:**
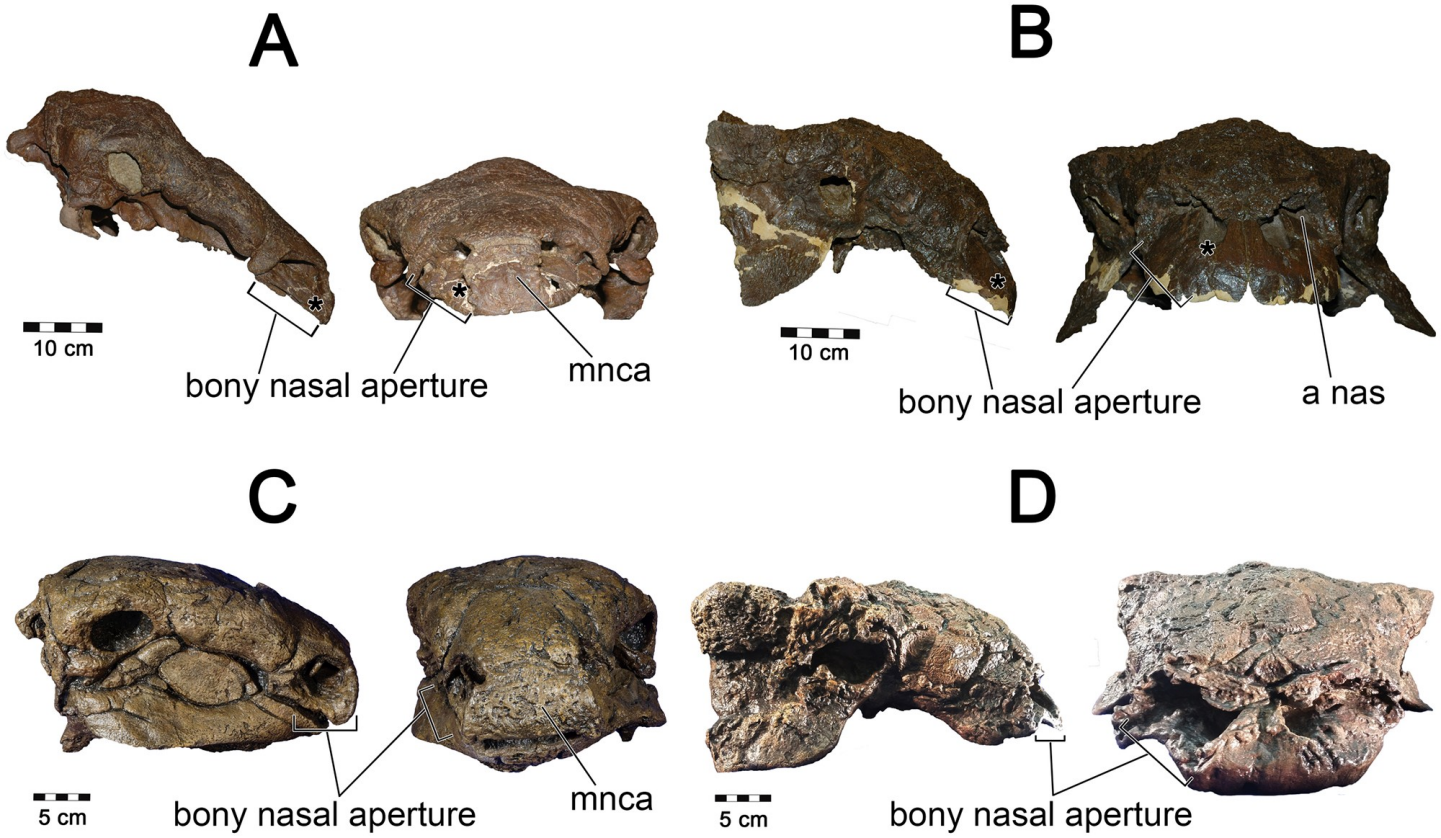
Nostril placement in ankylosaur models. **All skulls in right lateral and rostral views**. For *Panoplosaurus mirus* (A, C) we used (A) ROM 1215 as our base model with nostril placement informed by (C) CMN 8530. For *Euoplocephalus tutus* (B, D) we used (B) AMNH 5405 as our base model with the skull of (D) ROM 1930 informing us on the limits to the extent of the nostril. Asterisks in A and B denote location of fleshy nostril in our models.

For *Panoplosaurus*, we used the well-preserved skull of CMN 2759 to determine the location of the nostril. CMN 2759 preserved the rostral wall of the bony narial aperture, which was comprised of the rostral-most cranial osteoderm, most similar to the median nasal caputegulum of *Euoplocephalus* [112]. These anatomical structures strongly suggested that the nostril of *Panoplosaurus* deviated laterally (Fig 22A and 22C). Laterally-facing nostrils are common among diapsids and such a placement in *Panoplosaurus* was not unexpected.

For *Euoplocephalus*, AMNH 5405 did not present a well-preserved rostral-most portion of the cranium. To assist with nostril positioning, we compared bony narial aperture shape in AMNH 5405 with ROM 1930 (Fig 22B and 22D). In the latter, preservation of the rostral-most region of the cranium revealed an enlarged bony narial aperture that faced forward suggesting terminal nostril placement. However, the width of the bony narial aperture encompassed both the rostral-most portion of the cranium as well as a lateral portion of the cranium. Thus, it remains possible that the nostril in *Euoplocephalus* could have deviated laterally, or had a rostrolateral combination thereof. This position would be consistent with fleshy nostril placement in *Ankylosaurus* (AMNH 5214) where dermal ossification is extensive and indicates unambiguously that the nostril was located rostroventrolaterally [77]. In contrast, *Anodontosaurus lambei* (CMN 8530), a close relative of *Euoplocephalus*, shows a well constrained bony narial aperture that would limit the nostril to a terminal position on the snout [112]. All known skulls of *Euoplocephalus* that preserve the rostral-tip of the snout show a much less constrained bony narial aperture. This could indicate that the caputegula covering the cranium were less extensive in this species and that terminal fleshy nostrils were present but were constrained only by soft-tissues. AMNH 5405 has a fossa on the premaxillae (apertura nasalis [17]) that has previously been interpreted as a portion of the nasal vestibule ([17,20], Fig 21B and 21D). This fossa is wide enough that it could have easily housed a laterally deviating nasal vestibule that terminated rostrally in a laterally-facing nostril. For the purposes of our analysis we fit *Euoplocephalus* with such a nostril, with the caveat that the osteological evidence for it was equivocal (Fig 22B and 22D). Such a position is consistent with the general finding in amniotes that fleshy nostrils tend to be rostroventrally situated within the nasal vestibule [113].

#### Choana

**Fig 23:**
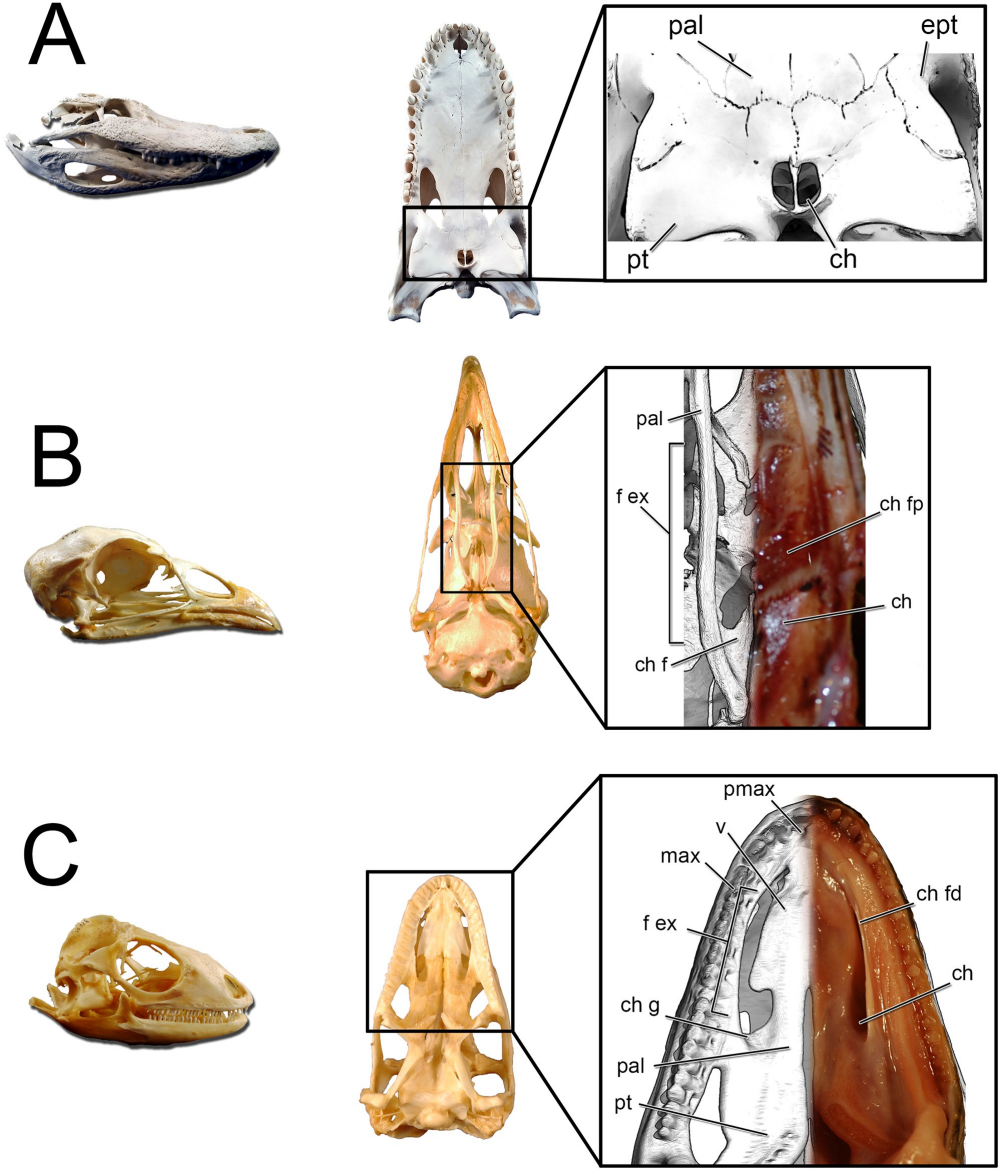
Lateral and ventral views of extant diapsid skulls illustrating the location of the choana. Crocodylians such as (A) *Alligator mississippiensis* (OUVC 9412) have a greatly retracted, apomorphic secondary choana. *Inset*: The bony boundaries to the secondary choana correspond to the soft-tissue boundaries. Birds such as (B) *Meleagris gallopavo* (OUVC 9647) retain the plesiomorphic placement of the choana. *Inset*: Magnified palatal region showing the difference between the bony boundaries to the choana (left side of image) and the soft-tissue boundaries (right side of image). Lizards such as (C) *Iguana iguana* (OUVC 10446) similarly show the plesiomorphic position of the choana. *Inset*: Relationship between the bony boundaries to the choana (left side of image) and the more restricted soft-tissue boundaries (right side of image).

The choana is the fleshy, “internal nostril” for the nasal passage. It represents the terminus of the airway within the nasal passage as the airway passes into the throat. Much as how the fleshy nostril resides within the larger narial fossa, the choana is typically associated with a much larger structure called the fenestra exochoanalis ([114,115] Fig 23) or bony choana [80]. The difference in shape between the fleshy and bony structures varies across species. In birds, the fenestra exochoanalis is extensive. It is bordered by the palatines laterally and caudally, the vomers medially, and the maxilla rostrally [115]. The choana opens as a fleshy slit at the caudal terminus of the fenestra exochoanalis in birds, and is often covered in life by “choanal flaps” that keep food particles out of the nasal passage during ingestion [116,117]. Osteologically, the choana is associated with a depression in the palatines referred to as the choanal fossa ([116], Fig 23B). Lizards have a choanal morphology similar to birds (Fig 23C). Their extensive fenestra exochoanalis is bounded by the maxillae rostrally and laterally, the palatines caudally and medially, and the vomers medially. As with birds, the choana resides at the caudal-most extent of the fenestra exochoanalis [118,119]. However, unlike birds the fleshy covering of much of the fenestra exochoanalis is less extensive and food appears to be prevented from entering the nasal passage partially by the more lateral placement of the choana on the oral roof along with a well-developed choanal fold that extends the majority of the length of the fenestra exochoanalis ([119], Fig 23C). As with birds, lizard choanae are associated with a choanal fossa (= choanal groove, [118,119]) situated at the caudal-most extent of the fenestra exochoanalis. Depending on the lizard species, the choana either opens or is greatly expanded in this region of the fenestra exochoanalis (Fig 23C). Crocodylians have an apomorphic choana placement referred to as the secondary choana [108]. It is produced via elongation of the nasopharyngeal duct through the palatines and into the pterygoids. The original or primary choana is still present and can be viewed internally within the dried skulls of extant crocodylians, where Witmer [108] observed it bounded medially by the vomer, caudally by the palatines and vomer, and laterally by the palatines and maxillae. These bony associations agree with choana placement in birds and lizards, thus suggesting that the primary choana is the location of the fenestra exochoanalis. The soft-tissue of the secondary choana is essentially an identical outline of the underlying bone, negating the need for a separate term for this region. Thus, the exit of the nasal cavity in crocodylians, regardless of soft-tissue presence, is the secondary choana (Fig 23A). Using the extant phylogenetic bracket approach (EPB, [120]), the presence of a choanal groove/fossa in both birds and lizards, can be considered a shared trait for diapsids that was later lost in crocodylians, making the choanal groove/fossa a level 1 inference for placement of the choana in the fenestra exochoanalis of diapsids [80].

**Fig 24:**
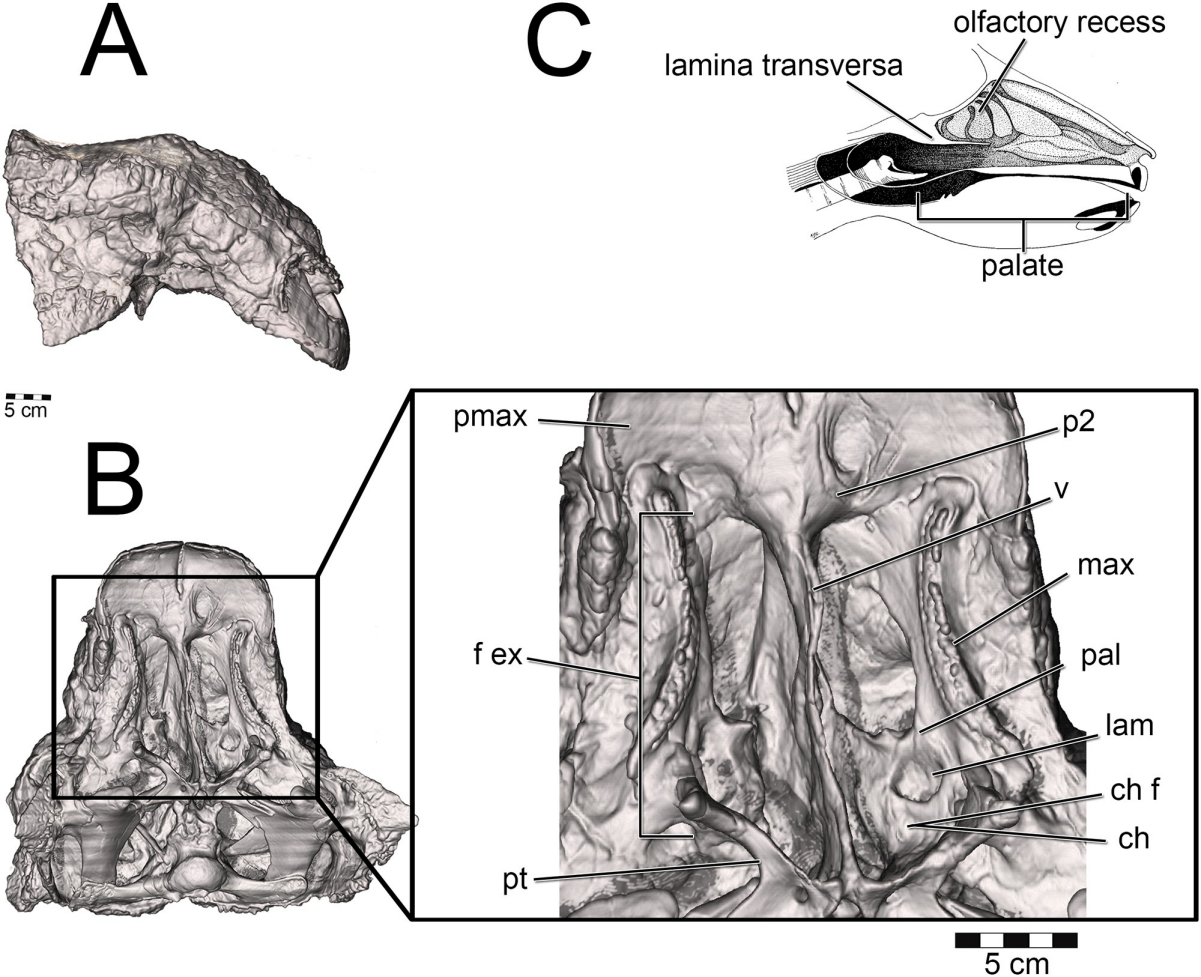
Palate identification and placement in *Euoplocephalus tutus* (AMNH 5405). (A) Skull in lateral and (B) ventral view. *Inset*: Major features of the palatal region. We refer to the caudodorsal secondary palate as equivalent to the lamina transversa observed in many mammals (C). Image in C modified from Cave [124].

**Fig 25:**
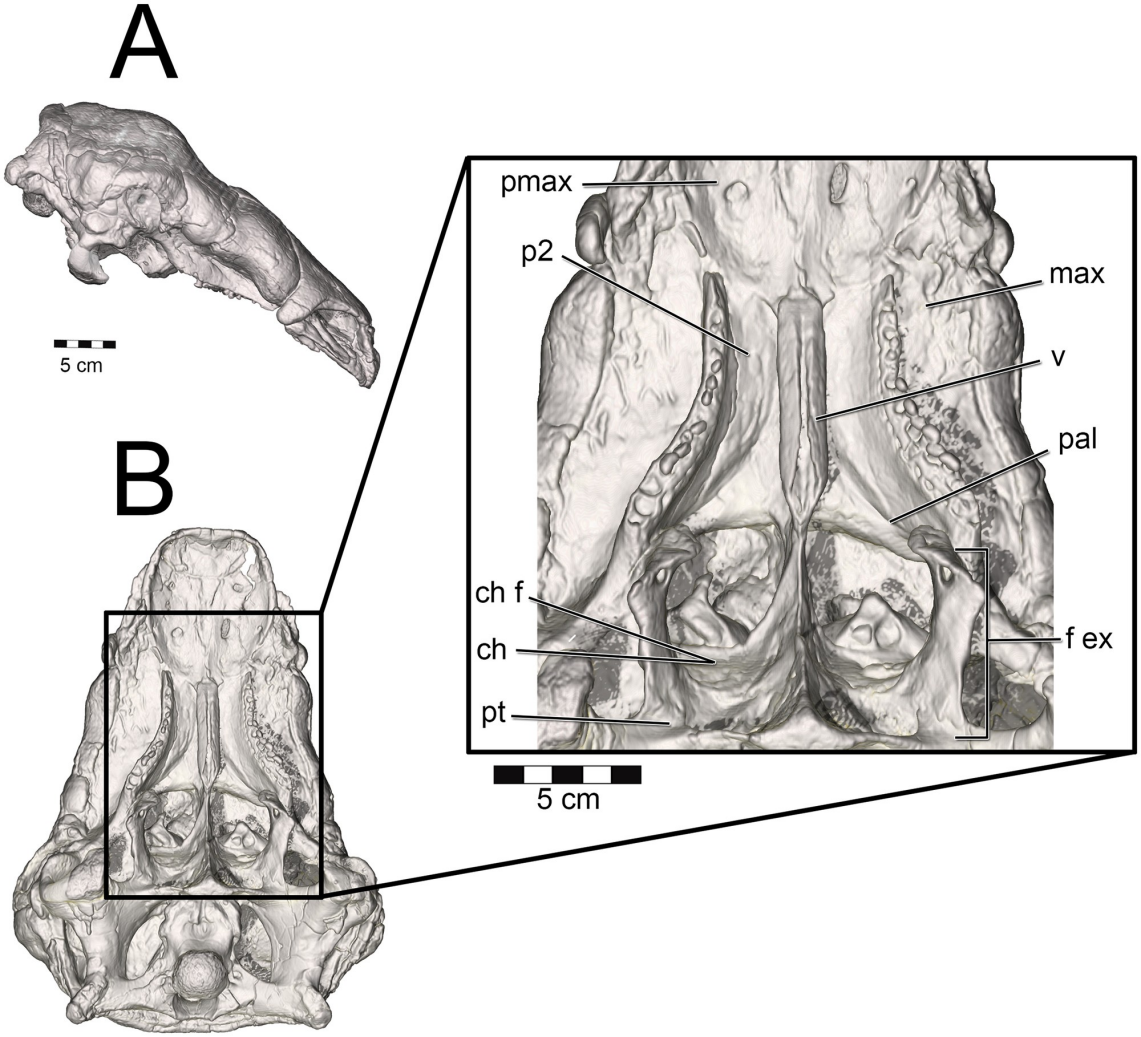
Palate identification and choana placement in *Panoplosaurus mirus* (ROM 1215). (A) Skull in lateral and (B) ventral view. *Inset*: Major features of the palatal region.

Both *Panoplosaurus* and *Euoplocephalus* had extensive fenestrae exochoanales (Figs 24 and 25). The shape of the soft palate in ankylosaurs has not been extensively studied. However, details on the hard-tissue anatomy indicate that despite an enlarged fenestra exochoanalis, there is evidence of bony secondary palate formation [1,13,17,121]. The secondary palate of ankylosaurs has traditionally been viewed as a bipartite structure [13]. Rostrally, elongated vomers contact the premaxilla, which sends out medial processes along with the maxilla to form palatal shelves, making a structure referred to as the “rostrodorsal secondary palate” [13,121]. Caudally, the palatines join with the vomers and pterygoids to form a structure called the “caudodorsal secondary palate” [13,17,121]. In light of new information on the shape of the nasal passage in ankylosaurs, the terminology for the palatal region of ankylosaurs should be revised. The rostrodorsal secondary palate in ankylosaurs such as *Euoplocephalus* should be viewed as the secondary palate (Fig 24), which is consistent with the usage of the term in other tetrapods in which the premaxillae and maxillae (and sometimes even palatines) meet rostral to the choanae [122]. The “caudodorsal secondary palate” serves to separate the olfactory recess from the rest of the nasal cavity. This structure is equivalent to the nasal structure known in mammals as the lamina transversa ([48,123,124], Fig 24C) and thus takes no part in the formation of the definitive palate. Crocodylians exhibit a similar partitioning of the nasal passages, with the roof of their nasopharyngeal duct forming the floor of their olfactory recess [108]. A large depression in the caudal aspect of the palatines appears equivalent to the choanal fossa or choanal groove seen in most extant diapsids (Figs 23 and 24). As such, we interpret this region as the opening of the choana into the throat. This interpretation makes the lamina transversa the bony boundary for an elongate nasopharyngeal duct. In nodosaurids such as *Panoplosaurus*, the distinction between the secondary palate and the choana is more evident [19,125], Fig 25). As with *Euoplocephalus*, there is evidence of a choanal fossa on the caudal aspect of the palatines, suggesting that the choana opened caudally in this taxon as well (Fig 25).

#### Soft-tissue correction

**Fig 26:**
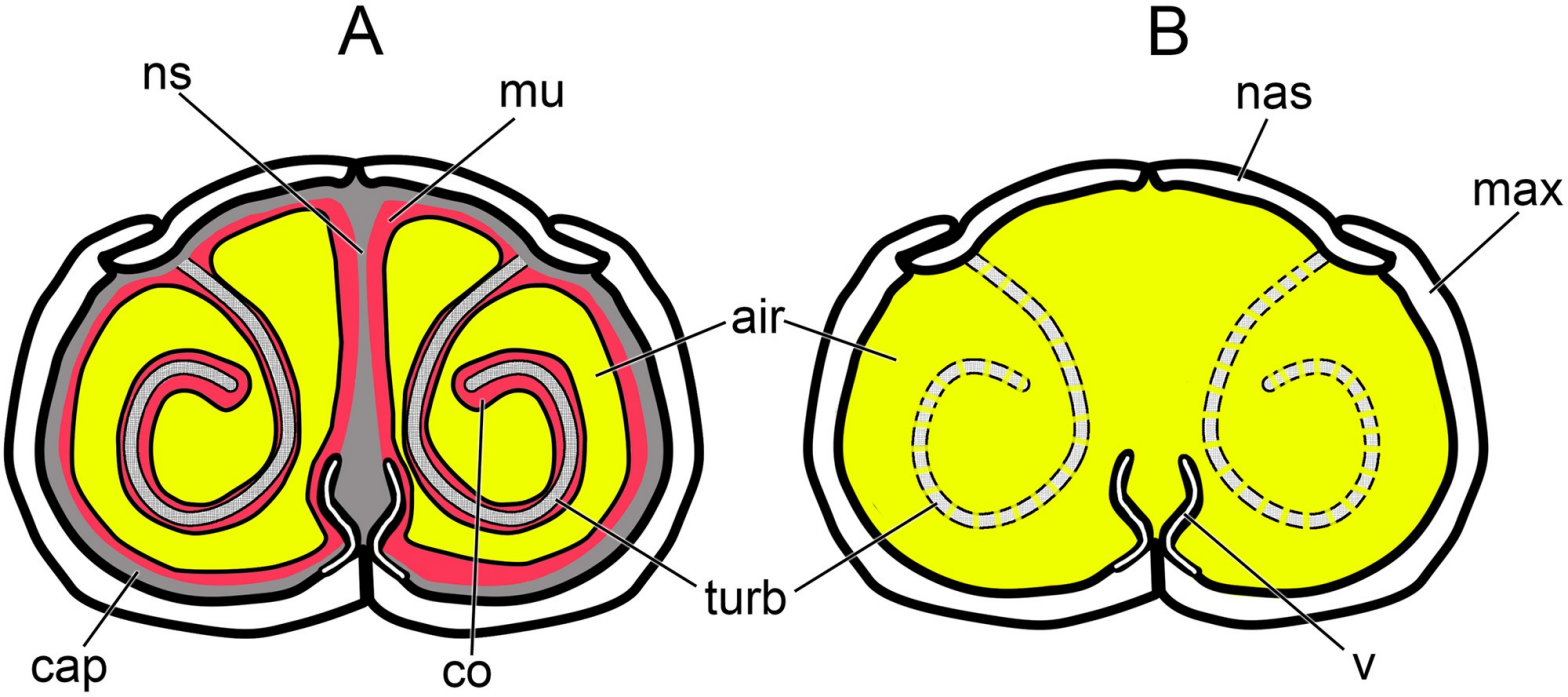
Generic airway diagram for diapsids. Note the much more constricted airway in the soft-tissue nasal passage (A) vs. the emptier, bony-bounded nasal passage typically preserved in fossils (B).

Airways segmented from the skulls represented the outer limits of the nasal passage, referred to here as the bony-bounded (BB) airway (Figs 15C and 16C). In life, soft tissues within the nasal passage such as the nasal capsule cartilages, mucosa, nerves, and vasculature would have been present and would have occupied space within these well-constrained nasal passages (Fig 26). Previous surveys of airway calibers in the nasal passages of extant amniotes found that airway caliber (the distance spanned between mucosal walls) does not exceed 10 mm in diameter regardless of the size (0.1–600kg) or phylogenetic position (from squamate reptiles to artiodactyl mammals) of the animal [33,80]. This constraint appears to be dictated by the biophysical limitations of diffusion, which effectively works across only very small distances [71]. The thermoregulatory and olfactory functions of the nasal cavity are both diffusion-dependent functions [29,31,126]. In contrast to data from extant animals, the average BB airway calibers in *Panoplosaurus* and *Euoplocephalus* were 15.8 mm and 22.9 mm, respectively. These were significantly larger calibers than that observed in the mucosa-lined airways of extant amniotes. To bring airway calibers within the range observed in extant amniotes, we imported airway models of *Panoplosaurus* and *Euoplocephalus* into the 3D modeling and animation program Maya (Autodesk, San Rafael, CA) where the airways were compressed using the program’s 3D deformation tools. Airway calibers were reduced until the average diameter was ~10 mm, which is the upper limit observed in extant amniotes, making this a conservative estimate for ankylosaurs. Airway compression followed the natural contours of the nasal passage such that the refined airways resembled a more compressed version of the original segmentation (Figs 15 and 16). These soft-tissue-corrected airways are here referred to as the ST airways (Figs 15D and 16D). An extension off the choana was added to all models tested. This extension served to replicate the connection of the nasal airway to the larynx and trachea. This extension was added to address technical aspects of the software (see below) and to ensure that fully developed airflow would be present at the choana during expiration, thus removing any potential heat flow artifacts created by having the program initialize airflow at the choana during expiration (Figs 15 and 16).

To further test the hypothesis that the convoluted airways in our two ankylosaur taxa were conferring a heat-transfer benefit, we digitally manipulated duplicates of our ST airways to remove either the length or the convolutions from the nasal vestibule (Figs 17 and 18). One version had a nasal vestibule that extended the straight-line distance between the nostril and the CNP. This model, referred to as the basic airway (Figs 17B and 18B), was used to represent what a simplified or plesiomorphic nasal vestibule would look like. The second version of the ST airway retained the total length of the nasal vestibule but had the curvature of the airway removed (Figs 17A and 18A). This model was referred to as the straightened airway. It represented the effects of airway distance alone on heat transfer through the nasal passage.

#### Boundary conditions

**Fig 27:**
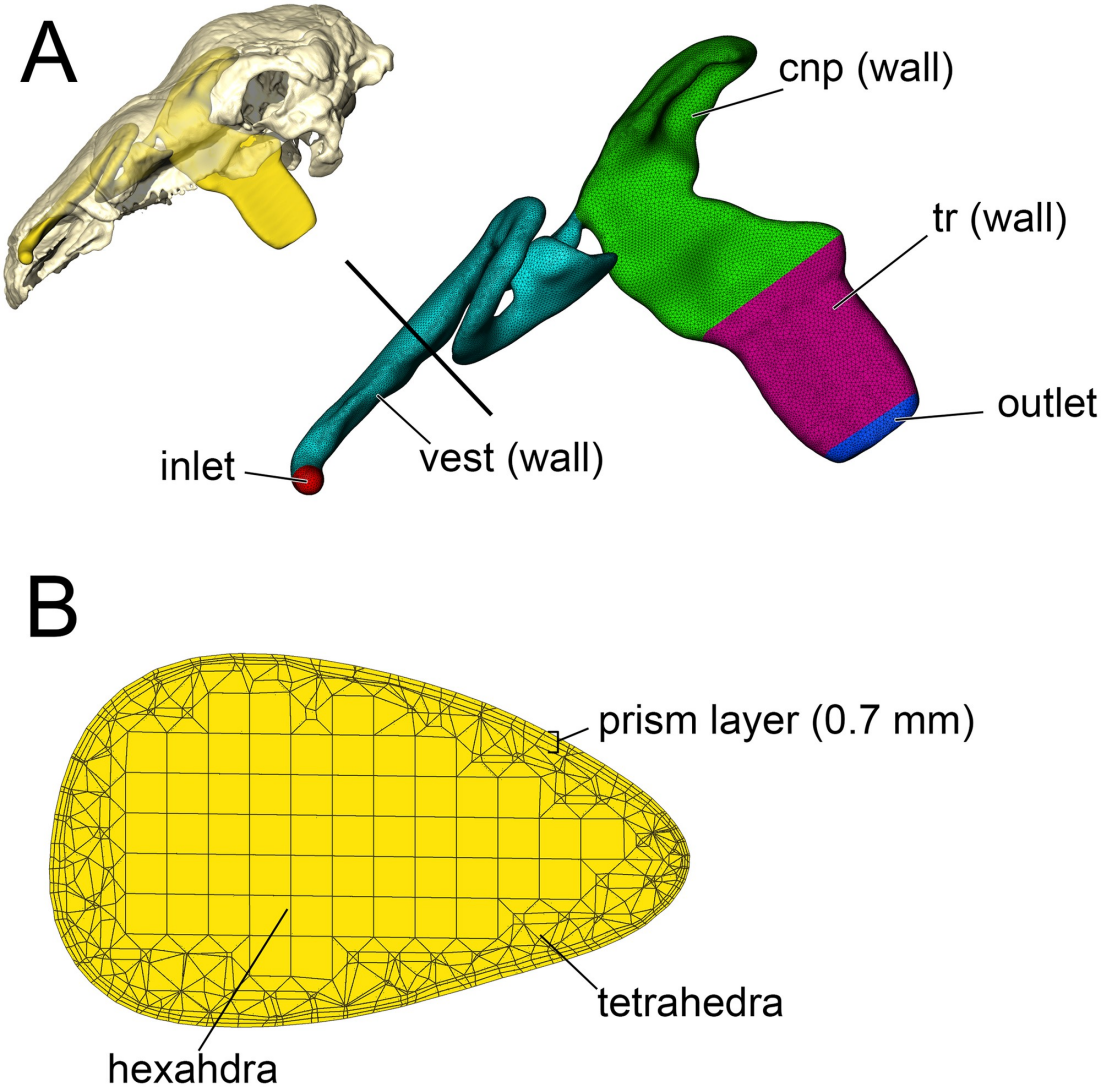
Mesh example for *Panoplosaurus mirus* (ROM 1215). (A) Nasal passage was assigned a series of boundary conditions (color-coded). Black line indicates location of (B) axial cross section illustrating the distribution of volumetric cells within the nasal passage.

Prior to volumetric meshing, the airway models were assigned a series of boundary conditions comprising a set of criteria that described this region of the model to the CFD program. Boundary condition assignment was done to elicit physiologically realistic airflow within the nasal passage (i.e., pressure driven air movement between nostril and choana). These conditions consisted of a pressure inlet located at the fleshy nostril and a pressure outlet located at the end of the artificial trachea (Fig 27A). During expiration, the assignment of these boundary conditions (inlet and outlet) was swapped. A series of impermeable wall boundaries covered the rest of the nasal passage model. Wall boundaries were demarcated based on anatomical location (Fig 27A). This was done to better control for regional variation in heat transfer across the nasal passage. Each wall boundary was considered rigid and incorporated a “no-slip” condition that states that air at the fluid-solid interface would be static, an assumption based on known properties of fluid movement through enclosed structures [71]. Note that amniote nasal passages do not have a truly rigid boundary layer between the mucosa and the air field. Boundary layers act as obstacles to diffusion-based processes, thus it is beneficial for amniotes to have means of reducing the size of these boundary layers. In extant amniotes, there is a mucociliary “conveyor belt” comprised of ciliated epithelium that beats in unison towards the nostril or choana [127,128]. This conveyor belt serves to move mucous across the mucosa of the nasal passage. This movement has the potential to reduce the boundary layer between the mucosa and the air field, which would aid in diffusion of heat and odorant molecules across the air-mucosa boundary. However, the speed of cilial movement is extremely slow (≤ 1 cm/min, [45]) compared to airflow, and its effects on airflow and heat transfer can be considered negligible for the purposes of our study. Thus, our use of a no-slip boundary condition should not hamper or otherwise reduce the quality of our results.

#### Meshing

Volumetric meshing was performed using the meshing program ICEM CFD (ANSYS Inc., Canonsburg, PA). Models consisted of a tetrahedral-hexahedral (tet-hex) hybrid mesh. First, an unstructured tetrahedral mesh was constructed using the robust OCTREE method [129]. After mesh reconstruction, the core of the mesh was deleted and flooded with hexahedra wherever possible. This hybrid construction offered the versatility of tetrahedra for unstructured mesh reconstruction [130,131], coupled with the computational efficiency of hexahedra [132,133]. To better resolve wall boundary effects on heat transfer, we incorporated a prism layer along the wall of the nasal passage. This layer consisted of four cells that grew in size from the wall by 1.5 times their parent cell, producing a combined thickness of approximately 0.7 mm (Fig 27B).

#### Computational fluid dynamic analysis

Meshes were imported into the CFD program Fluent (ANSYS Inc., Canonsburg, PA) for analysis. To determine the appropriate fluid dynamic model to apply to our airway models, we first took cross sections throughout the nasal passages to determine the dimensionless Reynolds and Womersley numbers for the airway. The Reynolds number is a staple of fluid dynamic analyses [71,134], representing the mathematical relationship between the viscous and inertial forces within a fluid. Low Reynolds numbers (< 2000) indicate that viscous forces dominate the system and that orderly (i.e, laminar) flow will be the dominant flow type expected. Reynolds numbers between 2000–4000 indicate a transition zone in which laminar flow may be punctuated with periods of turbulence that manifest in the form of secondary flows such as Von Kármán trails [71]. Reynolds numbers above 4000 indicate that inertial forces dominate the system and that flow will be chaotic or turbulent [71,134]. We used the following equation to calculate the Reynolds number for the airway [135]:

$$Re=\frac{4Q}{Pv}$$ (3)

where Q = volumetric flow rate (m^3^/sec), P = the wetted perimeter in meters [136], and v = the kinematic viscosity of air at 15°C (1.412e^-5^ m^2^/sec).

We used the dimensionless Womersley number [137] to determine the steadiness of the flow field, or how often airflow was able to produce a steady, parabolic flow profile. This profile is related to the size of the airway, viscosity of the fluid, and frequency of the oscillation (i.e., breathing rate). Womersley numbers < 1 indicate a quasi-steady flow field that can be modeled as time independent, or steady-state. As the Womersley number climbs above unity, steadiness decreases. At Womersley numbers > 10 the oscillation of the flow is too high for flow to completely develop [138] and is considered unsteady, thus requiring a transient or time-step-based modeling approach. We calculated the Womersley number using the following equation [45]:

$$Wo=\frac{Dh}{2}\sqrt{\frac{2\pi f}{v}}$$ (4)

where f = the frequency of oscillation (Hz), and Dh = hydraulic diameter of the airway. Hydraulic diameter was calculated using the following equation [136]:

$$h=\frac{4A}{P}$$ (5)

where A = the area of the cross section measured (m^2^).

#### Physiological variables

Following our previous methodology [80], we used the same phylogenetically corrected allometric equation for resting respiration in birds [49] to estimate flow rates during inspiration and expiration (Table 8). Mass estimates for both ankylosaurs were taken from the supplementary data in Brown et al. [139]. Estimated masses for the two ankylosaurs were over 20 times larger than the largest animal in the Frappell et al. dataset ([49], an 88 kg ostrich). Using the equations from Frappell et al. [49] required extending the regression line well beyond the initial scope of the data. To alleviate the effects of this approach we used the lightest mass estimates for *Panoplosaurus* and *Euoplocephalus* as provided by Brown et al. ([139], Table 8).

**Table 8:** Respiratory values for the ankylosaurs *Panoplosaurus mirus* and *Euoplocephalus tutus*.

Taxon	Mass (kg)*	Tidal volume (ml)†	Breathing frequency†	High flow rate†	Low flow rate‡
*Panoplosaurus*	1100	34,000	1.5 breaths/min	77 L/min	37 L/min
*Euoplocephalus*	2000	64,000	1.2 breaths/min)	110 L/min	48 L/min

*Brown et al. 2013. [73]

^**†**^Frappell et al. 2001. [49]

^‡^ Reversed-Reynolds approach.

We performed a cross-sectional analysis of the airways following the methodology of Bourke et al. [80]. Respiratory flow variables input into the Reynolds and Womersley equations above indicated that both taxa would have had steady flow, but air would have been largely transitional if not completely turbulent within the nasal passages during simulated respiration. The latter result was at odds with previously published results on resting respiration in amniotes. In extant amniotes measured during restful breathing, laminar flow dominates the air field [48,140–142]. Laminar flow is less energetically expensive (due to lower resistance) than turbulent flow and can be expected in animals that are not undergoing strenuous exercise. Our two ankylosaurs did not show this pattern, suggesting that the regression equations we used may not be viable at such large body masses or that ankylosaur body plans were not appropriate for the avian-based equation of Frappell et al. [49]. To reduce the effects of poor flow rate choice, we recalculated airflow rates by rearranging the Reynolds equation to solve for flow rate:

$$Q=\frac{(Re)Pv}{4}$$ (6)

We refer to this new method as the Reversed-Reynolds approach. This approach provides an upper limit to volumetric flow through the nasal passage under relaxed conditions. We set the Reynolds number at 2000 (corresponding to the transition into turbulence) as our constant and recalculated flow rate across the nasal passages. The lowest flow rate obtained from cross-sectional analysis using this Reversed-Reynolds equation was chosen as the new flow rate (Table 8). This approach, the first of its kind as far as we know, ensures that laminar flow dominates the air field under simulated respiration.

Flow rate has been shown to be the primary variable affecting the efficiency of the nasal passage at managing heat flow [29,70]. With this in mind, we chose to run the ST and BB models under both flow rate estimates. The initial flow rate, estimated from the regression by Frappell et al. [49], was deemed the “high flow rate” condition. Our revised flow rate estimate, calculated using our Reverse-Reynolds method, was deemed the “low flow rate” condition. For the high flow rate condition, we used the Wilcox two-equation κ–ω turbulence model [143] with low Reynolds corrections and shear stress transport. For the low flow rate condition, we used the standard laminar viscosity model for continuity, momentum, and energy.

$$\nabla ∙\underset{u}{\to }=0$$ (7)

$$\rho (\underset{u}{\to }∙\nabla )\underset{u}{\to }=\nabla \rho +\mu \nabla^{2}\underset{u}{\to }$$ (8)

$$(\underset{u}{\to }∙\nabla )T=\frac{k}{\rho C_{p}}\nabla^{2}T$$ (9)

Where $\underset{u}{\to }=\underset{u}{\to }(x,y,z)$ = the velocity vector, ρ = the density of air at a given temperature, T = T(x,y,z) temperature at a given time, Cp = the specific heat, and k = the thermal conductivity for air at a given temperature.

#### Environmental conditions

We simulated sea-level air at 15°C and 50% relative humidity (r.h.), which was within the range of expected conditions that these dinosaurs would have experienced in their environment [144,145]. We gave the nasal passages an estimated body temperature of 35°C. This body temperature fell within the range of core body temperatures typically observed in extant large terrestrial mammals, birds, and reptiles (Table 5). Density (1102 kg/m^3^), thermal conductivity (0.34 W/mK), and specific heat for the mucosal walls (3150 J/kgK) were obtained from the database provided by the Foundation for Research on Information Technologies in Society (IT’IS). Nasal walls were given a thickness of approximately 0.5 mm for heat to conduct through. This distance simulated the distance between the nasal passage and the adjacent blood vessels and was based on observed CT data from extant amniotes [146]. Humidity was simulated by using the species transport option in Fluent, which allows for the incorporation of the mass fractions of water at various temperatures (i.e., relative humidity). The nasal walls were assumed to be at 100% relative humidity during both phases of respiration, following observation on extant animals [147].

Pressure and velocity coupling used the SIMPLEC algorithm along with a node-based discretization gradient. We used a second order accurate spatial discretization scheme for pressure, momentum, turbulence (when applicable), and energy.

Models ran until the results obtained from each analysis had reached a specified level of stability and consistency referred to as convergence [148]. This indicated that the numerical process used to solve the problem had asymptotically approached the “true” solution (i.e., airflow and heat transfer through a real nasal passage) given the conditions provided to the program. In CFD, convergence may be determined based on the global imbalances in the values for each node within the mesh between steps, or iterations, of the model. Imbalances, or errors, between each iteration are referred to as residuals [148]. The smaller these residuals are, the smaller the error is and the more converged the solution becomes. Global variables for momentum, pressure, and continuity (the conservation of fluid mass) are generally considered solved when their residuals have fallen below 1.0e^-3^ [148]. However, for physiological studies such as ours, we applied the stricter criterion of 1.0e^-4^ [45,80]. For energy (heat flow), convergence is determined when the residuals of error had fallen below 1.0e^-6^. To further aid in determining convergence we monitored special surfaces placed throughout regions of the model. These surfaces were designed to output data from a single location only (point surfaces). They provided localized measures of convergence and were used in conjunction with standard convergence measures for continuity, momentum, and energy to determine when models had been sufficiently solved.

#### Mesh independence

**Fig 28:**
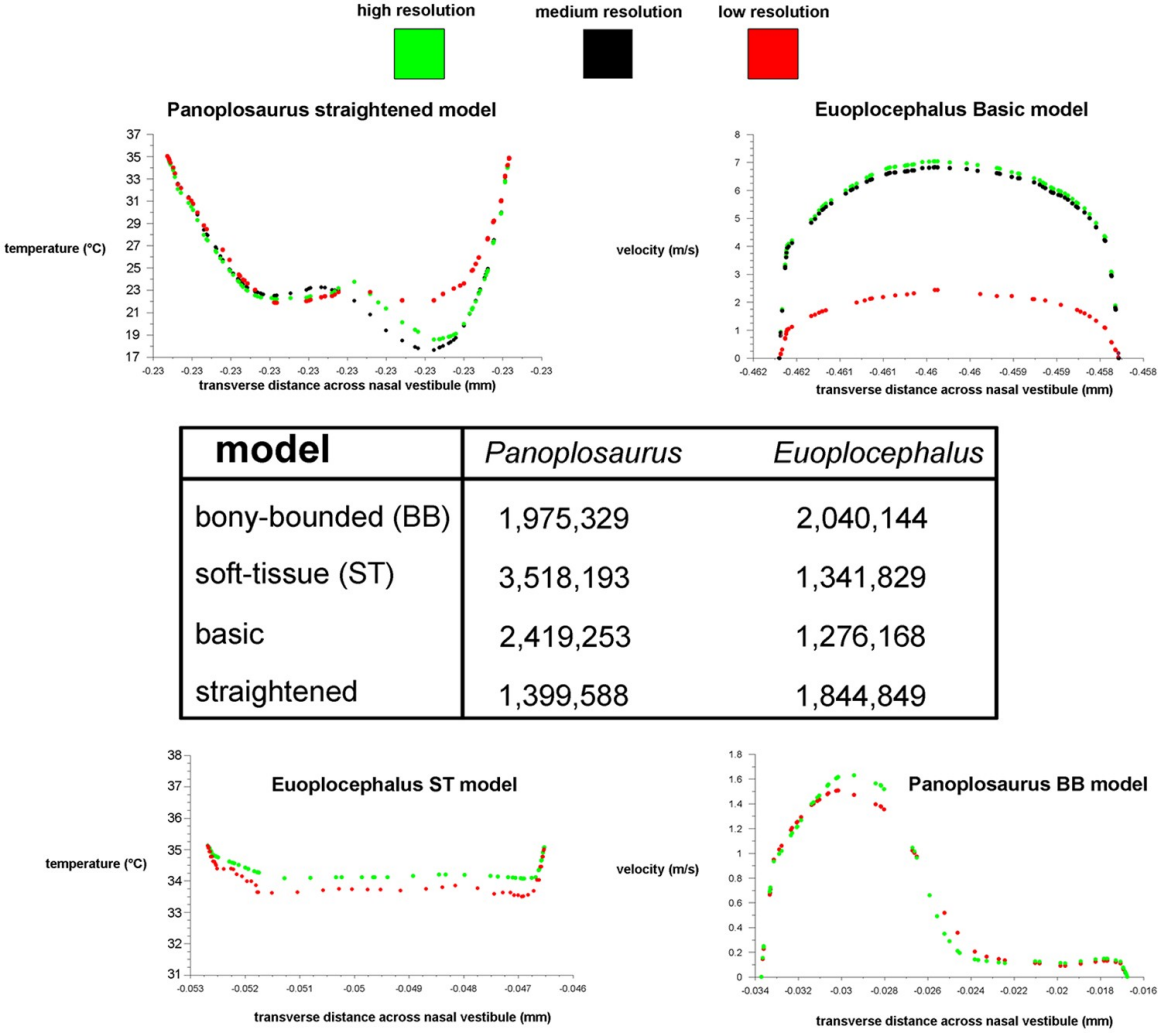
Final model resolutions used for simulations. Surrounding graphs show sample adaptive mesh comparisons between different model resolutions for temperature and velocity within parts of the nasal vestibule.

To ensure that the results obtained from our analyses were independent of the mesh resolution, we performed a solution-based adaptive mesh refinement (AMR), following previously established protocols [82]. This approach used converged or a mostly converged solution to determine regions of the mesh that had poor resolution. Local refinement was performed in these regions of poor resolution and the analysis was run again. This approach was repeated until refined meshes return values that fell below a pre-determined threshold. For our analysis, we used a threshold of 1% for regional temperature values as determined from point surfaces (Fig 28).

#### Post processing and heat flow measurements

Solved models were exported to the CFD module of Avizo (Avizo Wind) where qualitative and quantitative measurements were taken. For heat flow, we took measurements from cross sections of the nasal passage. These cross sections were taken orthogonal to the curvature of the nasal passage. Multiple measurements were taken from each cross section and the mean was recorded for each cross section.

#### Caloric costs and savings

Calculating the energetic costs of heating a single bolus of air by 20°C requires knowledge of the mass of air being moved between breaths, coupled with the caloric cost of heating that bolus of air. We calculated our estimated energetic costs using the following equation:

$$heating\;cost(cal)=(M_{air}*Cp)*\Delta T$$ (10)

where M_air_ = the mass of air in a given tidal volume (g), and Cp = the specific heat capacity of air, which is 0.24 cal/g°C across most physiological temperatures [147]. ΔT is the temperature change the air volume goes through (°C).

The mass of air present in a single breath was determined by multiplying the tidal volume of air respired, by its density at 35°C, as shown in the following equation:

$$M_{air}=V_{T}*1.146$$ (11)

where V_T_ = the tidal volume (L) and 1.146 = the density of air (g/L) at the estimated body temperature of 35°C. Body-temperature air density will be the limiting factor behind tidal volume within the lungs. To determine tidal volume, we used the equation relating tidal volume to body mass (M) in birds [49].

$$V_{T}={20.3M}^{1.06}$$ (12)

Much of the heat loss from the nasal passage occurs via evaporation of water off the nasal mucosa [147]. Thus, along with the caloric cost of temperature change within the nasal passage (sensible heat), one must also take into account the caloric cost associated with the phase change of water from a liquid to a gas (latent heat). We used saturated steam table values to determine the latent heat of vaporization for our temperature range. The caloric cost of heating air by 20°C was calculated using the following equation:

$${heating\;cost}_{\left(latent\right)}={\Delta M}_{H2O}∙{\Delta H}_{vap}$$ (13)

where ΔM_H2O_ = the absolute difference in the mass of water (kg) at two given humidities. ΔH_vap_ = the latent heat of vaporization for a given temperature (cal/kg). The mass of water was determined by multiplying the mass fraction of water at a given humidity (g/kg), by the mass of air (kg) in a single tidal volume.

As heat is a form of energy, the same equations for calculating the sensible heat gain to the air can be used to determine heat loss during expiration. Similarly, the caloric costs associated with the latent heat of vaporization will be the same values as the latent heat of condensation, just with a reversed sign. Thus, the equations used to determine the caloric costs of heating air during inspiration can be used to determine heat savings upon air cooling during expiration. The only change during expiration is the value for ΔH at the expired air temperature (cooler air holds less water), and the assumption that air leaves the nostril at 100% relative humidity regardless of expired air temperature [147].

Energy savings were calculated by taking the calories returned to the body during air cooling and condensation during expiration and dividing it by the initial caloric cost of heating and humidifying the air during inspiration. Water saving were calculated following the method of Schmidt-Nielsen et al. [149].

#### Heat savings comparison with extant taxa

Comparison of the results from our two ankylosaur species to extant animals was accomplished by surveying the literature for data on heat and water savings within the nasal passages of extant birds, mammals, and reptiles (Tables 9–11). Direct data was available for the cactus wren (*Campylorhynchus brunneicapillus*) and kangaroo rat (*Dipodomys merriami*).[29] All other studies reported only the estimated water recovery from respiration. We manually calculated the heat savings for the remaining species based on estimates of inspired air during a single breath. These estimates were obtained from mass-dependent equations for tidal volume in mammals [150] and lizards [85]. Estimated caloric costs and estimated savings were calculated using the methods described earlier (Tables 9 and 10).

**Table 9:** Taxa used for comparative energy savings graph.

Taxon	Reference
*Campylorhynchus brunneicapillus*	[29]
*Dipodomys merriami*	[29]
*Giraffa camelopardalis*	[33]
*Equus africanus*	[33]
*Dipsosaurus dorsalis*	[30]
*Corvus brachyrhynchos*	[38]
*Columba livia*	[38]

**Table 10:** Inspiratory values for taxa studied.

Taxon	Tidal volume (ml)	Mass of air (g)	Heat capacity (cal/°C)	Temperature increase (°C)	Total energy (cal)
*Giraffa camelopardalis* [33]	5,959	6.78	1.63	16.2	26.4
*Equus africanus* [33]	1,605	1.82	0.44	14	6.16
*Dipsosaurus dorsalis* [30]	0.457	5.12e^-4^	1.23e^-4^	12	1.5e^-3^
*Corvus brachyrhynchos* [38]	7.25	8.14e^-3^	1.95e^-3^	26.1	5.1e^-2^
*Columba livia* [38]	5.24	5.90e^-3^	1.41e^-3^	25.7	3.6e^-2^

**Table 11:** Heat energy savings among taxa studied.

Taxon	Expired temp (°C)	Mass of air (g)	Heat capacity (cal/°C)	Temp decrease (°C)	Energy saved (cal)
*Giraffa camelopardalis* [33]	28.0	6.98	1.7	9.3	15.8
*Equus africanus* [33]	32.3	1.86	0.45	5.3	2.4
*Dipsosaurus dorsalis* [30]	35	5.24e^-4^	1.3e^-4^	7	9.1e^-4^
*Corvus brachyrhynchos* [38]	21.9	8.68e^-3^	2.1e^-3^	19.2	4.0e^-2^
*Columba livia* [38]	21.4	6.28e^-3^	1.5e^-3^	19.3	3.0e^-2^

The results produced from this method provide a rough, “ballpark” comparison between taxa. They allowed us to see the overall energy recovery capacity within the nasal passages. However, since these data came from three different studies with different protocols, these comparative results should not be viewed as equivalent. For instance, the study on the desert iguana (currently the only reptile to have such a study done) had measurements of inspiration and expiration at a variety of body temperatures [30]. To make the results from that paper more comparable to the mammal and bird data, we took the largest exhaled temperature drop observed, which was 7°C when the animal had a body temperature of 42°C in an ambient temperature of 30°C (note: Murrish and Schmidt-Nielsent [30] had a typographical error in their discussion that stated their lizards reduced air temperature by only 5°C. However, their results section and graphs indicate that 7°C is the correct number). Further, the authors did not test their lizards at an ambient temperature of 15°C, nor a relative humidity of 50%, thus making direct comparisons with Langman et al. [33] and Geist [38] impossible. Similarly, the data from Schmidt-Nielsen et al. [29] were for animals breathing in air at 25% relative humidity, whereas the data from Langman et al. [33] did not specify humidity, nor did they test at air temperatures of 15°C. Of the data used for comparisons, the data from Geist [38] are the most equivalent for comparison with our dinosaur models.

#### Validation study

Prior to running our analysis on the ankylosaur models, we sought first to validate our methodology using empirically obtained data on heat transfer in pigeons [38]. We used CT data from a large adult domestic pigeon (*Columba livia*) and followed the methodology outlined above to simulate heat transfer within the nasal passage (Table 1, Fig 3). As with the ankylosaurs, we only modeled heat transfer through the left nasal passage. Data obtained during inspiration were used to inform the expiration model under the assumption that warming of the air by the nasal walls came at the expense of an equal reduction in mucosal wall temperature. Environmental temperature and humidity were set to 15°C and 50% relative humidity, respectively, reflecting the conditions used by Geist [38].
